# Vitamin D Intake, Serum 25-Hydroxyvitamin-D (25(OH)D) Levels, and Cancer Risk: A Comprehensive Meta-Meta-Analysis Including Meta-Analyses of Randomized Controlled Trials and Observational Epidemiological Studies

**DOI:** 10.3390/nu15122722

**Published:** 2023-06-12

**Authors:** Mehmet Emin Arayici, Yasemin Basbinar, Hulya Ellidokuz

**Affiliations:** 1 Department of Preventive Oncology, Institute of Health Sciences, Dokuz Eylul University, 15 July Medicine and Art Campus, Inciralti-Balcova 35340 İzmir, Türkiye; 2 Department of Translational Oncology, Institute of Oncology, Dokuz Eylul University, 35340 İzmir, Türkiye; ybaskin65@gmail.com; 3 Department of Biostatistics and Medical Informatics, Faculty of Medicine, Dokuz Eylul University, 35340 İzmir, Türkiye; hulyazeyda@gmail.com; 4 Department of Preventive Oncology, Institute of Oncology, Dokuz Eylul University, 35340 İzmir, Türkiye

**Keywords:** cancer, meta-analysis, vitamin D, mortality, incidence

## Abstract

It is a well-established fact that inadequate Vitamin D (Vit-D) levels have negative effects on the development and progression of malignant diseases, particularly cancer. The purpose of this paper was to elucidate the effects of Vit-D intake and serum 25-hydroxyvitamin-D (25(OH)D) levels on cancer incidence and mortality, the current evidence in this field, and the biases of this evidence, using the meta-meta-analysis method. Meta-analyses focusing on Vit-D intake, serum 25(OH)D levels, and cancer risk/mortality were identified. A structured computer literature search was undertaken in PubMed/Medline, Web of Science (WoS), and Scopus electronic databases using predetermined keyword combinations. Primary and secondary meta-meta-analyses were carried out, combining odds ratios (ORs), risk ratios (RRs), and hazard ratios (HRs) for outcomes reported in selected meta-analyses. A total of 35 eligible meta-analyses (59 reports yielded from these studies) assessing the association between Vit-D and cancer incidence and/or mortality were included in this study. In the pooled analysis, higher Vit-D intake and serum 25(OH)D levels were associated with lower cancer risk (OR = 0.93, 95% confidence interval (CI): 0.90–0.96, *p* < 0.001; OR = 0.80, 95% CI: 0.72–0.89, *p* < 0.001, respectively) and cancer-related mortality (RR = 0.89, 95% CI: 0.86–0.93, *p* < 0.001; RR = 0.67, 95% CI: 0.58–0.78, *p* < 0.001, respectively). When meta-analyses whose primary reports included only randomized controlled trials were pooled, there was no significant association between Vit-D intake and cancer risk (OR = 0.99, 95% CI: 0.97–1.01, *p* = 0.320). In subgroup analysis, Vit-D consumption was associated with a significant decrease in colorectal and lung cancer incidence (OR = 0.89, 95% CI: 0.83–0.96, *p* = 0.002; OR = 0.88, 95% CI: 0.83–0.94, *p* < 0.001, respectively). Taken together, both Vit-D intake and higher 25(OH)D levels may provide remarkable benefits in terms of cancer incidence and mortality; however, careful evaluation according to cancer types is critically important and recommended.

## 1. Introduction

Cancer, which is a complicated group of malignant disorders characterized by abnormal cell proliferation and an uncontrolled cell cycle, remains a major cause of death globally, regardless of human development levels, in countries all over the world [1,2,3,4]. According to Global Cancer Observatory (GLOBOCAN) data, approximately 19.3 million new cancer cases were reported, and 10 million deaths were attributed to cancer worldwide in 2020 [2,3]. Based on the 2019 data provided by the World Health Organization (WHO), cancer is identified as the primary or secondary cause of death for individuals under the age of 70 in over half of the world’s nations (183 countries). In other countries, it ranks as the third or fourth leading cause of mortality [2,5].

Vitamin D (Vit-D) was identified as a pro-hormone that provides a range of health benefits, from bone health to immune function, and plays critical roles in biological processes in human metabolism [6,7]. It is widely acknowledged that inadequate Vit-D levels have adverse effects on the development and advancement of malignant disorders, mainly cancer, because they impair immune adequacy and increase the risk of complications. Therefore, it has the potential to influence the general well-being and quality of life (QoL) of individuals, directly or indirectly [8,9,10]. Observational epidemiological studies on Vit-D have emphasized the importance of Vit-D in both preventing cancer and cancer-related deaths and improving the prognosis of patients with cancer [8,9,10,11].

As is well documented, numerous epidemiological studies have reported outcomes suggesting a potential correlation between Vit-D insufficiency and the risk of cancer, as well as cancer-related deaths [12,13,14,15,16,17]. The findings from these studies have provided compelling evidence for the relationship between higher Vit-D levels and a decreased risk of acquiring cancers, including breast, colon, prostate, and lung cancer [15,16,17]. Increasing evidence strongly suggests that maintaining adequate Vit-D levels may play a protective and pivotal role in reducing the risk of numerous types of carcinoma. However, conflicting outcomes have been reported in previous primary studies and meta-analyses regarding serum 25-hydroxyvitamin-D (25(OH)D) levels, Vit-D intake, and cancer development and progression. According to this point of view, in a comprehensive meta-analysis conducted by Goulão et al. [18], consisting of randomized controlled trials (RCTs) involving 18,808 participants, the results demonstrated no substantial evidence supporting the notion that Vit-D intake alone contributes to a reduction in cancer incidence or cancer-related deaths. This conclusion remained consistent even after incorporating long-term follow-up data (risk ratio (RR) = 1.03, 95% CI: 0.91–1.15, *p* > 0.05). Similarly, two recent meta-analyses evaluating Vit-D intake and cancer risk reported that Vit-D intake did not decrease the overall cancer incidence (RR = 0.99, 95% CI: 0.94–1.04, *p* > 0.05; RR = 0.98, 95% CI: 0.94–1.02, *p* > 0.05) [19,20]. In contrast to the previously specified investigations, several meta-analyses that incorporated observational epidemiological studies found an association between high Vit-D intake or serum 25(OH)D levels and a decreased risk of different types of cancer. Some of these meta-analyses reported that increased serum 25(OH)D levels or higher Vit-D intake were associated with a decreased risk of colorectal cancer [12,14,21]. Furthermore, several reports of studies have suggested a lower risk of liver cancer [13], ovarian cancer [22], and lung cancer [23,24] among individuals with higher Vit-D intake or higher levels of serum 25(OH)D.

In general, studies related to the potential benefits of Vit-D intake and serum 25(OH)D statuses in decreasing the risk of cancer and cancer-related mortality are important topics of ongoing research and discussion in the scientific society. In addition, it is a well-known fact that meta-analyses examining the association between Vit-D intake, serum 25(OH)D levels, and different forms of cancer hold considerable significance due to the high level of evidence they provide to the scientific community. In the last decade, there has been a notable enhancement in the number of published meta-analyses that specifically evaluate the relationship between Vit-D and cancer. An additional crucial point for consideration is the ongoing necessity to consistently reevaluate and consolidate the existing evidence regarding the potential advantages or disadvantages of Vit-D in order to decrease the risk of cancer and cancer-related mortality. The major purpose of this reevaluation is to assess the current state of the epidemiological landscape, which has evolved extensively over the course of time.

Therefore, the primary objective of this study was to elucidate the impact of Vit-D intake and serum 25(OH)D levels on cancer incidence and cancer-related mortality utilizing the meta-meta-analysis method. Additionally, we aimed to comprehensively assess the existing evidence in this field and identify any potential biases among the published meta-analyses.

## 2. Methods

Meta-analyses that specifically considered the relationship between Vit-D intake, serum 25(OH)D levels, and cancer risk and/or cancer-related mortality were identified for inclusion in the study. In accordance with this purpose, to ensure methodological rigor and transparency, the study strictly adhered to the standardized methodology guidelines recommended in the Preferred Reporting Items for Systematic Reviews and Meta-Analysis (PRISMA) [25] and Meta-analysis of Observational Studies in Epidemiology (MOOSE) [26] guidelines. These guidelines provided a comprehensive framework for conducting the study, ensuring consistent and reliable methods at all stages of the meta-meta-analysis. The PRISMA Checklist was associated with Supplementary Table S1. This checklist served as a tool to verify compliance with the PRISMA guidelines and to ensure the completeness and accuracy of the reporting in the study.

PICOs:

1. Population: “Patients with cancer and individuals without cancer”
2. Intervention: “Vit-D intake and serum 25(OH)D levels”
3. Comparison: (i) “Low and high Vit-D intake”; (ii) “low and high serum 25(OH)D levels”
4. Outcomes: (i) “Cancer risk”; (ii) “Mortality”
5. Study: “Systematic reviews with meta-analysis or meta-analysis alone”

### 2.1. Search Strategy and Data Extraction

A structured computer literature search was undertaken in PubMed/Medline, Web of Science (WoS), and Scopus electronic databases using predetermined keyword combinations. Keyword selection was structured by considering three main factors: “cancer”, “vitamin D” and “meta-analysis”. Once the search strategy was formulated through the Pubmed/Medline database, it was adapted to other databases (WoS and Scopus). Medical Subject Headings (MeSH) and text terms were incorporated into the search strategy via Boolean operators (AND/OR). Keyword combinations were identified as follows: “vitamin D” (Title/Abstract) OR “D vitamin” (Title/Abstract) OR “calciferol” (Title/Abstract) OR “cholecalciferol” (Title/Abstract) OR “cholecalciferol-D3” (Title/Abstract) OR “Vitamin-D3” (Title/Abstract) OR “25 hydroxy vitamin D” (Title/Abstract) OR “25 hydroxy vitamin D3” (Title/Abstract) AND “cancer” (All Fields) OR “tumor” (All Fields) OR “neoplasms” (All Fields) OR “tumors” (All Fields) “malignance” (All Fields) AND “meta-analysis” (Title/Abstract). Details of the algorithms used for the three databases (Pubmed/Medline, WoS, and Scopus) are illustrated in Supplementary Table S2.

### 2.2. Selection Criteria

**Figure 1:**
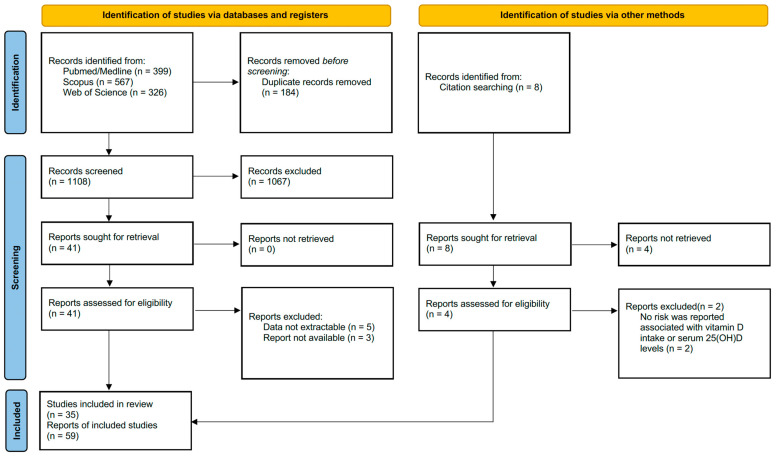
The PRISMA flowchart illustrating the relevant literature search and the study selection process.

Initially, a preliminary data review was conducted to assess the suitability of systematic reviews (with meta-analysis) concerning the questions and objectives of the research. During the initial assessment, the title, abstract, and keywords of each meta-analysis were thoroughly scrutinized. This evaluation process involved carefully reviewing the provided information to determine the relevance of the meta-analysis to the research question or topic of interest. If the abstracts contained insufficient information, the full text was examined. In the second evaluation, a detailed examination of the full texts was performed to determine whether the studies fulfilled all the inclusion criteria. The data illustrated in the results section were extracted using a structured protocol that was specifically designed to capture the most applicable information from each study [27]. The PRISMA flowchart showing the selection process for included and excluded studies is available in Figure 1.

Meta-analyses reporting a risk in terms of incidence and/or mortality associated with cancer and Vit-D intake (low and high intake) or serum 25(OH)D levels (low and high level) were included in the study. The study exclusively considered reports and papers that were published in English and were available in full text. Animal model experiments, cell culture studies, non-original publications (letter to the editor, case report), systematic reviews without meta-analysis, and outcomes not reported as risks (odds ratios (ORs), risk ratios (RRs), or hazard ratios (HRs)) have been excluded from the study. The results reported in each meta-analysis were synthesized by two independent and qualified investigators (MEA and HE). Data extracted from each study were processed in a predefined and structured Microsoft Excel^®^ spreadsheet. After removing all data from the meta-analyses included in the research by two independent investigators, the other researcher (YB) independently reviewed and cross-checked the data to ensure accuracy, consistency, and reliability, in order to reach a consensus. Any discrepancies or inconsistencies that arose were thoroughly discussed, evaluated, and resolved through consensus among the research team.

### 2.3. Methodological Quality Assessment

The quality of meta-analyses was evaluated utilizing the 16-item AMSTAR-2 (A MeaSurement Tool to Assess Systematic Reviews) tool (Table S3) [28]. Seven of the sixteen items in AMSTAR-2 were classified as crucial items (items 2, 4, 7, 9, 11, 13, 15). AMSTAR-2 has been defined as an evaluation tool, developed to enable the evaluation of systematic reviews of randomized and non-randomized studies of health interventions. AMSTAR-2 was not intended to constitute an overall score. Each item was evaluated as “yes”, “partial yes” or “no” according to the standard. The overall evaluation of studies (high, moderate, low, or critically low) was based on the evaluation of critical and non-critical items. The quality of the included meta-analyses was also evaluated by two independent researchers.

### 2.4. Assessment of Risk of Bias

The Risk of Bias in Systematic Reviews (ROBIS) (Table S4) [29] tool was used to assess the risk of bias in the included papers. The ROBIS tool is designed to assess the risk of bias in systematic reviews and/or meta-analyses. The risk of bias is assessed in three phases. In the first phase, “assessment relevance” is evaluated. The aim of the second phase is to “identify concerns with the review process”. In the third phase, a comprehensive evaluation related to “data collection and study appraisal” is presented.

### 2.5. Data Appraisal, Synthesis, and Statistical Analysis

Primary and secondary meta-meta-analyses were carried out combining OR, RR, and HR for outcomes reported in selected meta-analyses. Initially, an analysis was performed that summarized all existing data into a single pooled estimate. After initial pooling, subgroup analyses were executed to evaluate the heterogeneity of outcomes and to examine the effects of Vit-D intake and serum 25(OH)D status in different cancer and study types. Pooled effect sizes (ES) and ORs, RRs and/or HRs were calculated at 95% confidence intervals (CI) for all groups in primary and secondary meta-meta-analyses. A predefined spreadsheet was created using Microsoft Excel^®^ to systematically document key qualitative and quantitative data from the incorporated studies.

Egger’s linear regression asymmetry (statistical significance set at *p* < 0.10) test [30], schematic illustration of the funnel plots, and Begg and Mazumdar’s rank correlation test [31], which provided the z-value for Kendall’s tau, were utilized to calculate the potential for publication bias. In instances where publication bias was detected, the trim and fill method was employed to adjust for this bias [32]. The heterogeneity of the outcomes from the different studies was evaluated using the χ2-based Cochran’s Q test (*p* < 0.05) and *I*^2^ statistics (percentage of variances in the effect estimates due to statistical heterogeneity). The *I*^2^ statistics describe the observed percentages based on the variance in the true effects. In the assessment of *I*^2^ values, a result of 25% indicated low heterogeneity, 50% indicated moderate heterogeneity, and 75% indicated high heterogeneity, adhering to established conventions in the field of evidence synthesis [33]. In the statistical calculations of primary and secondary meta-meta-analyses, method selection was performed taking into account heterogeneity among the studies. When significant heterogeneity was detected among the studies, analyses were performed utilizing the random effects model. If there was no significant heterogeneity, analyses were carried out via a fixed effects model. Statistical significance in all meta-meta-analyses was quantified at the two-tailed *p* < 0.05 level. Meta-meta-analysis statistical calculations were performed employing Prometa3^®^ [34], along with the R statistical software version 4.2.0 [35], following established guidelines for meta-analytic procedures.

### 2.6. Sensitivity Analysis

The robustness of the outcomes was evaluated through sensitivity analyses. In the sensitivity analyses, each individual study was excluded separately from the pooled analysis to assess its impact on the overall results, and then the change in ES was examined. Studies reporting outliers are identified by this method. When necessary, these studies are excluded from the pooled analysis and the confidence intervals are strengthened.

### 2.7. Mapping

A graphical representation in the form of a bubble chart was generated for each systematic review, allowing for a visual depiction of the scientific evidence and facilitating an understanding of the information. The review information was structured and illustrated using a three-dimensional approach as follows:

1. Study population (bubble size and bubble color): The size of each bubble is structured to be directly proportional to the sample size of the original studies included in each of the systematic reviews. Moreover, studies with a relatively large sample, studies with a medium sample, and studies with a relatively small sample were colored separately.

2. Impact (x) axis: The meta-analyses were categorized and organized based on their respective publication years, providing a chronological framework.

3. Strength of results (y-axis): The structure of the analysis was based on the number of primary studies included in each review, providing a quantitative representation of the research encompassed by each study.

## 3. Results

### 3.1. Search Results

A total of 1292 papers were identified in the preliminary scans of the relevant databases (Pubmed/Medline (*n* = 399), Scopus (*n* = 567), and WoS (*n* = 326)), and 184 of these studies were removed without review due to duplicate records. After the duplicate records were removed, the studies (*n* = 1108) yielded were evaluated by examining the titles and abstracts. In this preliminary review, 1067 ineligible pieces of research were excluded from the study. The full texts of 49 papers were evaluated in detail, including 41 articles in the main search and 8 articles in the citation search. After all reviews, 59 reports from 35 papers [12,13,14,18,19,20,21,22,23,24,36,37,38,39,40,41,42,43,44,45,46,47,48,49,50,51,52,53,54,55,56,57,58,59,60] that ultimately met the inclusion criteria were incorporated in the meta-meta-analysis. The included (Table S6) and excluded meta-analyses and the reasons for excluding the removed papers are summarized in Table S7. The PRISMA flowchart showing the relevant literature scans and the study selection procedure is also demonstrated in Figure 1.

### 3.2. Baseline Characteristics of the Meta-Analyses

A total of 35 eligible meta-analyses [12,13,14,18,19,20,21,22,23,24,36,37,38,39,40,41,42,43,44,45,46,47,48,49,50,51,52,53,54,55,56,57,58,59,60] examining the association between Vit-D and cancer incidence and/or mortality and reporting risk for cancer incidence and/or mortality were included in the current study. The sample sizes of the studies varied between 1902 and 1,566,662. Of these meta-analyses, 20 studies [12,13,14,18,21,22,36,37,40,42,44,45,46,47,48,53,54,57,59,60] reported risk for total cancer and various cancer types, three studies [49,50,52] reported mortality, and 12 studies [19,20,23,24,38,39,41,43,51,55,56,58] reported both mortality and risk. In eight meta-analyses [18,19,20,39,41,56,58,60], the primary studies consisted of RCTs. One of these meta-analyses reported breast cancer-related risk [60], while other studies documented total cancer incidence and/or cancer-related deaths. The primary reports of three meta-analyses [46,49,50] consisted of cohort studies. In other studies, case-control, cohort, and/or RCTs were evaluated in various combinations. The baseline characteristics of the papers comprised in the study are given in Table 1.

Methodological quality assessment of 35 meta-analyses was performed using the AMSTAR-2 tool (Table S3). In the vast majority of evaluated studies, two or more critical defects were identified (especially item 7). Therefore, it was observed that most of the meta-analyses did not have very high-quality scores. Detailed assessment results are shown in Supplementary Table S3. All included systematic reviews with meta-analysis were considered low risk in phase 1 and domain 1 according to ROBIS guidelines. We observed that there was no obvious risk of bias in most of the studies. In other domains also, there was no obvious risk of bias in most studies. Detailed assessment results are summarized in Table S5.

### 3.3. Outcomes

The primary meta-meta-analysis included 59 reports from a total of 35 eligible studies [12,13,14,18,19,20,21,22,23,24,36,37,38,39,40,41,42,43,44,45,46,47,48,49,50,51,52,53,54,55,56,57,58,59,60] evaluating Vit-D and cancer incidence/mortality. Vit-D intake and cancer risk were documented in 25 reports; serum 25(OH)D levels and cancer risk were documented in 18 reports; Vit-D intake and cancer-related mortality were documented in eight reports; and serum 25(OH)D levels and cancer-related mortality were documented in eight reports (Table 1).

### 3.4. Vitamin D Intake and Cancer Risk/Mortality

**Figure 2:**
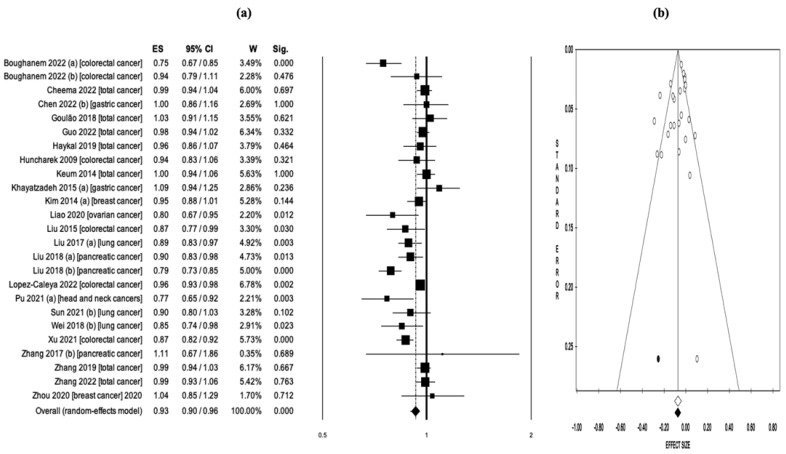
(**a**) Pooled effect size (ES) associated with vitamin D intake (low and high intake) and cancer risk, and (**b**) funnel plot [18,19,20,21,22,23,24,36,39,40,41,42,43,46,47,48,51,53,54,55,56,58,60].

A pooled analysis of a total of 25 reports evaluating Vit-D intake and cancer risk concluded that higher Vit-D intake was associated with lower cancer risk (OR = 0.93, 95% CI: 0.90–0.96, *p* < 0.001) (Figure 2a). Significant heterogeneity was detected among studies (Q = 85.1, *df* = 24, *I*^2^ = 71.8%, *p* < 0.001), and analyses were conducted utilizing the random effects model. The study reports did not demonstrate any evidence of publication bias, as indicated by the results of Egger’s linear regression asymmetry test (intercept = −1.05, t = −1.55, *p* = 0.134) and Begg and Mazumdar’s rank correlation test (z = −1.26, *p* = 0.207) (Figure 2b). The results of the sensitivity analysis, as shown in Figure S1, confirmed the consistency and reliability of the findings.

**Figure 3:**
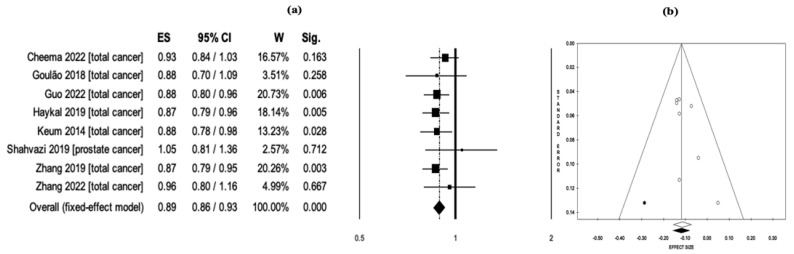
(**a**) Pooled effect size (ES) associated with vitamin D intake (low and high intake) and cancer-related mortality, and (**b**) funnel plot [18,19,20,39,41,52,56,58].

In a pooled analysis of a total of eight meta-analyses evaluating Vit-D intake and cancer-related mortality, higher Vit-D intake was associated with lower mortality (RR = 0.89, 95% CI: 0.86–0.93, *p* < 0.001) (Figure 3a). The heterogeneity analysis indicated no significant heterogeneity among the included studies (Q = 3.45, *df* = 7, *I*^2^ = 0.0%, *p* = 0.840). Hence, the meta-meta-analysis was conducted employing the fixed effects model. The evaluation of the funnel plot did not demonstrate any evidence of publication bias among the included studies in the analysis (Figure 3b). The sensitivity analysis provided validation of the robustness of the analysis results.

### 3.5. Serum 25-Hidroxyvitamin-D Levels and Cancer Risk/Mortality

**Figure 4:**
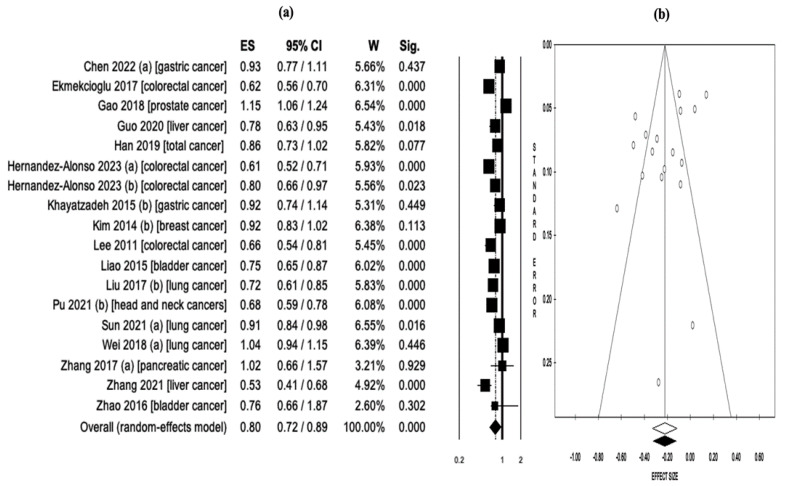
(**a**) Pooled effect size (ES) associated with serum 25(OH)D levels (low and high levels) and cancer risk, and (**b**) funnel plot [12,13,14,23,24,36,37,38,42,43,44,45,51,53,55,57,59].

A pooled analysis of a total of 18 reports assessing serum 25(OH)D levels and cancer risk found that higher serum 25(OH)D levels were associated with lower cancer risk (OR = 0.80, 95% CI: 0.72–0.89, *p* < 0.001) (Figure 4a). Considerable heterogeneity was observed among the included studies (Q = 164.3, *df* = 17, *I*^2^ = 89.6%, *p* < 0.001). Therefore, analyses were executed utilizing a random effects model. The study reports showed no evidence of publication bias based on the results of Begg and Mazumdar’s rank correlation test (z = −0.87, *p* = 0.384) (Figure 4b). The sensitivity analysis conducted in this synthesis confirmed the stability and reliability of the test results (Figure S2). 

**Figure 5:**
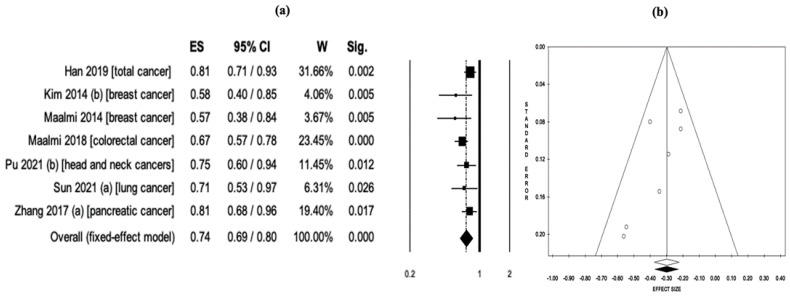
(**a**) Pooled effect size (ES) associated with serum 25(OH)D levels (low and high levels) and cancer-related mortality, and (**b**) funnel plot [24,38,43,49,50,51,55].

In a pooled analysis of eight meta-analyses examining the relationship between serum 25(OH)D levels and cancer-related mortality, it was found that higher serum 25(OH)D levels were associated with a 33% reduction in mortality (RR = 0.67, 95% CI: 0.58–0.78, *p* < 0.001). However, among the studies included in the analysis, a meta-analysis [23] reported a very low risk, thus creating a negative outlier in the analyses. The results of Egger’s linear regression asymmetry test (intercept = −3.09, t = −2.33, *p* = 0.059) and Begg and Mazumdar’s rank correlation test (z = −2.23, *p* = 0.026) suggested that there may be publication bias in a study’s report. Therefore, this study [23] was excluded from the analysis. In a re-pooled analysis of seven studies, it was observed that higher serum 25(OH)D levels were associated with a 26% reduction in mortality risk (RR = 0.74, 95% CI: 0.69–0.80, *p* < 0.001), as depicted in Figure 5a. No significant heterogeneity was detected between studies (Q = 7.67, *df* = 6, *I*^2^ = 21.7%, *p* = 0.263), and the meta-meta-analysis was performed using the fixed effects model. The study reports showed no evidence of publication bias, as indicated by the results of Egger’s linear regression asymmetry test (intercept = −1.95, t = −2.01, *p* = 0.101) and Begg and Mazumdar’s rank correlation test (z = −1.65, *p* = 0.099) (Figure 5b). The robustness of the analysis results was further confirmed by sensitivity analyses, which demonstrated consistent findings and supported the reliability of the results.

### 3.6. Subgroup Analysis

In order to measure the sensitivity of the analyses and the robustness of the results, subgroup analyses were carried out in terms of Vit-D intake (low and high intake) and serum 25(OH)D levels (low and high levels). 

In the secondary meta-meta-analyses, subgroup analyses were performed according to study types (RCTs and observational) and cancer types. Meta-meta-analyses were conducted if there were at least three studies for different cancer types in the subgroup analyses. Accordingly, in the pooled analysis of studies evaluating Vit-D intake and total cancer risk, it was observed that Vit-D intake did not cause a remarkable change in cancer risk (OR = 0.99, 95% CI: 0.97–1.01, *p* = 0.300) (Table 2). In subgroup analyses of colorectal and lung cancer, Vit-D intake was associated with a significant reduction in cancer risk (OR = 0.89, 95% CI: 0.83–0.96, *p* = 0.002; OR = 0.88, 95% CI: 0.83–0.94, *p* < 0.001, respectively). The relationship between Vit-D intake and cancer mortality was evaluated with data from a total of seven reports. Accordingly, it was concluded that Vit-D intake was associated with a significant reduction in total cancer mortality (RR = 0.89, 95% CI: 0.85–0.93, *p* < 0.001).

A meta-meta-analysis was conducted to investigate the relationship between serum 25(OH)D levels and two specific types of cancer. As seen in Table 2, serum 25(OH)D levels were associated with a non-significant reduction in the incidence of lung cancer (OR = 0.89, 95% CI: 0.75–1.05, *p* = 0.178). In colorectal cancer, the analysis results strongly suggested that higher serum 25(OH)D levels were associated with a lower risk of colorectal cancer (OR = 0.65, 95% CI: 0.60–0.70, *p* < 0.001) (Table 2).

Meta-analyses whose primary reports included only RCTs were also pooled in subgroup analyses. Accordingly, there was no significant association between Vit-D intake and cancer incidence (OR = 0.99, 95% CI: 0.97–1.01, *p* = 0.320). However, there was a significant association between Vit-D intake and an 11% reduction in cancer-related mortality (RR = 0.89, 95% CI: 0.85–0.93, *p* < 0.001) as shown in Table 2.

### 3.7. Mapping

A visual map was created for the systematic reviews to visually display the study information via a bubble chart. Review information was evaluated in three dimensions. Bubble size varies in direct proportion to the sample size included in the study. The publication years of the meta-analyses are included in the effect (x) axis. The y-axis indicates the number of primary studies that were selected and included in the related meta-analyses. Studies with relatively large samples, studies with medium samples, and studies with relatively small samples are colored separately. The bubble chart associated with the mapping of the meta-analysis of 32 from 35 studies on Vit-D is presented in Figure S3. Three studies [36,40,44] were not included in the visual map because the sample size was not clearly reported.

## 4. Discussion

It is established that Vit-D deficiency and inadequate serum 25(OH)D levels are important risk factors for many cancers [8,12,13,14]. Many epidemiological studies have shown an inverse association between Vit-D levels and many types of cancer, including breast, prostate, colon, and lung cancer [15,16,17]. Our analysis suggests that there may be strong associations between Vit-D intake, serum 25(OH)D levels, and cancer risk, especially cancer-related mortality. Although most of the studies identified in our meta-meta-analysis (27 of 35 studies) included observational (cohort and/or case-control) studies, a combined evaluation of multiple meta-analyses yielded strong evidence. We supported the results with subgroup analyses in order to examine the differences in terms of study types.

The most recent meta-analyses [14,19,20,21,36,48,58] included in our study were documented in the literature in 2022 and 2023. Three of these studies included meta-analyses of RCTs [19,20,58], and primary reports of other studies included observational epidemiological studies [14,21,36,48]. Meta-analyses of RCTs in these most recent studies reported no notable variation between Vit-D intake and total cancer risk (RR = 0.99, 95% CI: 0.94–1.04, *p* > 0.05; RR = 0.98, 95% CI: 0.94–1.02, *p* > 0.05; RR = 0.99, 95% CI: 0.93–1.06, *p* > 0.05) [19,20,57]. In observational studies, however, two meta-analyses conducted by Hernandez-Alonso et al. [14] and Lopez-Caleya et al. [48] revealed an inverse relationship between serum 25(OH)D levels or Vit-D intake and the risk of colorectal cancer (OR = 0.61, 95% CI: 0.52–0.71, *p* < 0.05; OR = 0.96, 95% CI: 0.93–0.98, *p* < 0.05). Boughhanem et al. [21], on the other hand, reported that Vit-D intake was associated with a lower risk of cancer in case-control studies (OR = 0.75, 95% CI: 0.67–0.85, *p* < 0.05), while this association was not confirmed in prospective cohort studies (OR = 0.94, 95% CI: 0.79–1.11, *p* > 0.05). Similar to these current meta-meta-analyses, we found no significant difference between Vit-D intake and total cancer risk in pooled analyzes of RCTs in our study (OR = 0.99, 95% CI: 0.97–1.01, *p* = 0.320). However, in a pooled analysis of observational studies, intake of Vit-D was associated with a 10% lower risk of cancer (OR = 0.90, 95% CI: 0.86–0.95, *p* < 0.001).

The results of current primary studies and meta-analyses regarding serum 25(OH)D levels and Vit-D intake have revealed conflicting reports. In particular, meta-analyses focusing on RCTs have reported no remarkable evidence of a significant association between Vit-D intake and cancer. For example, in a meta-analysis of RCTs by Goulão et al. [18] that involved 18,808 participants, it was reported that there was no evidence that Vit-D intake alone reduced cancer incidence or cancer deaths, even after long-term follow-up results were included (RR = 1.03, 95% CI: 0.91–1.15, *p* > 0.05). Similarly, two recent (2022) meta-analyses evaluating Vit-D intake and cancer risk suggested that Vit-D intake did not reduce the overall cancer incidence (RR = 0.99, 95% CI: 0.94–1.04, *p* > 0.05; RR = 0.98, 95% CI: 0.94–1.02, *p* > 0.05) [19,20]. Contrary to these results, a meta-analysis executed by Han et al. [38] in 2019 that included prospective cohort studies provided evidence that higher serum 25(OH)D concentrations are marginally associated with lower cancer incidence and mortality (RR = 0.86, 95% CI: 0.73–1.02, *p* < 0.05; RR = 0.81, 95% CI: 0.71–0.93, *p* < 0.05, respectively). Similarly, in many meta-analyses that included observational epidemiological studies, high Vit-D intake or high serum 25(OH)D levels have been associated with a reduced risk of several types of cancer, such as colorectal [12,14,21], liver [13], ovarian [22], and lung cancer [23,24]. Similar results were emphasized in the literature in meta-analyses of RCTs evaluating Vit-D intake and cancer risk. While these studies revealed that there was no significant reduction in cancer risk with Vit-D intake, it was reported that Vit-D intake was associated with a significant decrease in cancer-related mortality [18,19,20,39,41,55,58,60]. Meta-analyses of observational epidemiological studies provided evidence of an inverse relationship between Vit-D and cancer risk [12,14,21,45,46,48]. Similar to the literature, in this study, we also concluded that higher Vit-D intake was associated with lower cancer risk in a pooled analysis of a total of 25 reports evaluating Vit-D intake and cancer risk (OR = 0.93, 95% CI: 0.90–0.96, *p* < 0.001). However, when only meta-analyses of RCTs were included in the pooled analysis, there was no significant association between Vit-D intake and cancer incidence. The majority of the studies included in our research (77.1%) were also observational studies. Therefore, based on these findings, it can be inferred that these results can be attributed to the data gathered from observational studies included in this research.

Another critical issue to address and discuss is the investigation of the reasons behind the discrepancies in findings observed between RCTs and observational studies. One considerable reason for the differences is that the primary endpoint in most of the primary studies included in the meta-analyses of RCTs did not focus on cancer incidence or cancer-related death. Furthermore, another contributing factor to the discrepancies between RCTs and observational studies may be that the participants included in the RCTs were not specifically selected from groups known to have a higher risk of Vit-D deficiency. Hence, due to the absence of participants specifically at higher risk for Vit-D deficiency in the RCTs, a notable effect of Vit-D intake may not have been observed in this group. Additionally, the differences in the specific dosing protocols employed in RCTs versus observational studies contribute to the differences between the findings of these two types of studies. Furthermore, another significant factor is that the majority of RCTs did not measure serum 25(OH)D levels at the conclusion of the study to evaluate the actual impact of Vit-D. Therefore, it is crucial to thoroughly consider and take into account these confounding factors when interpreting and comparing the results comparing RCTs and observational studies.

In a pooled analysis of a total of 18 reports evaluating serum 25(OH)D level and cancer risk, we observed that higher 25(OH)D levels were associated with lower cancer risk (OR = 0.80, 95% CI: 0.72–0.89, *p* < 0.001). This result also suggested that serum 25(OH)D levels are a better indicator for cancer risk. A total of seven meta-analyses assessing serum 25(OH)D levels and cancer-related mortality were pooled and analyzed. Accordingly, the results of the analysis revealed that higher serum 25(OH)D levels were associated with 26% lower mortality (RR = 0.74, 95% CI: 0.69–0.80, *p* < 0.001). Similarly, in a pooled analysis of a total of eight meta-analyses evaluating Vit-D intake and cancer-related mortality, higher Vit-D intake was associated with 11% lower mortality (RR = 0.89, 95% CI: 0.86–0.93, *p* < 0.001). These results also confirmed that serum 25(OH)D levels are a better indicator for cancer-related mortality.

In subgroup analyses, it was found that Vit-D intake did not significantly reduce or increase total cancer risk (OR = 0.99, 95% CI: 0.97–1.01, *p* = 0.300), whereas Vit-D intake was associated with a significant decrease in cancer risk in colorectal and lung cancer (OR = 0.89, 95% CI: 0.83–0.96, *p* = 0.002; OR = 0.88, 95% CI: 0.83–0.94, *p* < 0.001, respectively). Our results are compatible with the literature [14,18,19,21,23,40] and provide a high level of evidence. Although a meta-meta-analysis of RCTs showed that Vit-D intake was not associated with a reduction in cancer risk, the results of this study suggest that Vit-D intake and high serum 25(OH)D levels can significantly reduce the incidence and mortality of various cancers. Vit-D intake and high serum 25(OH)D levels may be associated with cancer risk and survival.

It is widely acknowledged that public health policies are formulated based on the evaluation of systematic reviews and meta-analyses, which are considered to provide the highest level of evidence. The present meta-meta-analysis has remarkably raised the level of evidence by incorporating numerous systematic reviews (with meta-analysis) and reassessing analyses with increased power. It also simplified the researcher’s task of evaluating these studies together, as it gathered the meta-analyses examining Vit-D intake, serum 25(OH)D levels, and cancer risk/mortality under one umbrella. Although this paper provides valuable evidence, it has several limitations that are worth considering. One limitation of this paper is the possibility of variations in patient selection and treatment protocols across the primary studies included in the meta-analyses. This could lead to heterogeneity across the studies, potentially affecting the overall conclusions. Another limitation of this investigation is the lack of an evaluation of the impact of the treatments received by cancer patients. The effect of treatments, such as chemotherapy and radiation therapy, on the relationship between Vit-D intake and cancer mortality, was not considered. Furthermore, the primary reports included in the meta-analyses within this study selected patients from various countries and geographical regions, which could lead to variations in Vit-D status and cancer incidence/mortality rates due to differences in diet, lifestyle, and other factors. This could impact the generalizability of the study’s conclusions to different populations.

## 5. Conclusions

In conclusion, this meta-meta-analysis of meta-analyses provided strong evidence indicating a significant association between Vit-D intake and serum 25(OH)D levels with cancer incidence and mortality. Taken together, both Vit-D intake and higher 25(OH)D levels may provide significant benefits in terms of cancer incidence and mortality, but careful evaluation on the basis of cancer types is recommended. Furthermore, it is crucial to implement accurate confounding controls in future research, particularly RCTs. Future research should place emphasis on enhancing study designs, incorporating larger sample sizes, implementing more precise confounding controls, and exploring the potential dose–response relationship between Vit-D intake and oncology outcomes. Continual evaluation of the evidence is critical in assessing the changing epidemiological landscape in studies of Vit-D and cancer, as well as in providing a solid basis for medical guidelines and clinical decision-making. The findings of this study may provide a solid basis for individual decision-making regarding Vit-D in the context of cancer.

## Figures and Tables

**Table 1:** The baseline characteristics of studies on vitamin D scrutinized in the research.

First Author/Year	Cancer Type	Characteristics of the Primary Studies	Vit-D Exposure	Total Number of Studies (*n*)	Total Sample Size (*n*)	Outcome	NoP Studies Included for Incidence (*n*)	NoP Studies Included for Mortality (*n*)	Effect Size (ES) and Confidence Interval (CI) for Incidence	Effect Size (ES) and Confidence Interval (CI) for Mortality
Boughanem 2022 (a) ^x^ [21]	Colorectal cancer	Case-control, prospective cohort	Vit-D intake	31	926,237	Incidence	12	N/A	OR = 0.75 (0.67–0.85)	N/A
Boughanem 2022 (b) ^y^ [21]	Colorectal cancer	Case-control, prospective cohort	Vit-D intake	31	926,237	Incidence	6	N/A	HR = 0.94 (0.79–1.11)	N/A
Cheema 2022 [19]	Total cancer	RCTs	Vit-D intake	13	109,543	Incidence, mortality	12	7	RR = 0.99 (0.94–1.04)	RR = 0.93 (0.84–1.03)
Chen 2022 (a) [36]	Gastric cancer	Case-control, prospective cohort	Serum 25(OH)D	11	N/A	Incidence	11	N/A	OR = 0.93 (0.77–1.11)	N/A
Chen 2022 (b) [36]	Gastric cancer	Case-control, prospective cohort	Vit-D intake	11	N/A	Incidence	4	N/A	OR = 1.00 (0.86–1.16)	N/A
Ekmekcioglu 2017 [12]	Colorectal cancer	Case-control, prospective cohort	Serum 25(OH)D	14	12,110	Incidence	14	N/A	RR = 0.62 (0.56–0.70)	N/A
Gao 2018 [37]	Prostate cancer	Case-control, prospective cohort	Serum 25(OH)D	19	48,369	Incidence	19	N/A	RR = 1.15 (1.06–1.24)	N/A
Goulão 2018 [18]	Total cancer	RCTs	Vit-D intake	30	18,808	Incidence	24	7	RR = 1.03 (0.91–1.15)	RR = 0.88 (0.70–1.09)
Guo 2020 [13]	Liver cancer	Case-control, prospective cohort	Serum 25(OH)D	6	60,811	Incidence	6	N/A	RR = 0.78 (0.63–0.95)	N/A
Guo 2022 [20]	Total cancer	RCTs	Vit-D intake	26	121,529	Incidence, mortality	19	11	RR = 0.98 (0.94–1.02)	RR = 0.88 (0.80–0.96)
Han 2019 [38]	Total cancer	Prospective cohort	Serum 25(OH)D	23	170,618	Incidence, mortality	8	16	RR = 0.86 (0.73–1.02)	RR = 0.81 (0.71–0.93)
Haykal 2019 [39]	Total cancer	RCTs	Vit-D intake	10	79,055	Incidence, mortality	9	5	RR = 0.96 (0.86–1.07)	RR = 0.87 (0.79–0.96)
Hernandez-Alonso 2023 (a) [14]	Colorectal cancer	Case-control	Serum 25(OH)D	28	140,112	Incidence	11	N/A	OR = 0.61 (0.52–0.71)	N/A
Hernandez-Alonso 2023 (b) [14]	Colorectal cancer	Prospective cohort	Serum 25(OH)D	28	140,112	Incidence	6	N/A	HR = 0.80 (0.66–0.97)	N/A
Huncharek 2009 [40]	Colorectal cancer	Case-control, cohort	Vit-D intake	60	N/R	Incidence	10	N/A	RR = 0.94 (0.83–1.06)	N/A
Keum 2014 [41]	Total cancer	RCTs	Vit-D intake	4	45,151	Incidence, mortality	4	3	RR = 1.00 (0.94–1.06)	RR = 0.88 (0.78–0.98)
Khayatzadeh 2015 (a) [42]	Gastric cancer	Case-control, cohort	Vit-D intake	7	59,626	Incidence	4	N/A	OR = 1.09 (0.94–1.25)	N/A
Khayatzadeh 2015 (b) [42]	Gastric cancer	Case-control, cohort	Serum 25(OH)D	7	59,626	Incidence	3	N/A	OR = 0.92 (0.74–1.14)	N/A
Kim 2014 (a) [43]	Breast cancer	Case-control, cohort	Vit-D intake	30	762,859	Incidence	12	N/A	RR = 0.95 (0.88–1.01)	N/A
Kim 2014 (b) [43]	Breast cancer	Case-control, cohort	Serum 25(OH)D	30	762,859	Incidence, mortality	14	4	RR = 0.92 (0.83–1.02)	RR = 0.58 (0.40–0.85)
Lee 2011 [44]	Colorectal cancer	Case-control, cohort	Serum 25(OH)D	8	N/A	Incidence	8	N/A	OR = 0.66 (0.54–0.81)	N/A
Liao 2015 [45]	Bladder cancer	Case-control, cohort	Serum 25(OH)D	5	89,610	Incidence	5	N/A	RR = 0.75 (0.65–0.87)	N/A
Liao 2020 [22]	Ovarian cancer	Case-control, cohort	Vit-D intake	29	963,604	Incidence	6	N/A	RR = 0.80 (0.67–0.95)	N/A
Liu 2015 [46]	Colorectal cancer	Cohort	Vit-D intake	47	870,330	Incidence	17	N/A	RR = 0.87 (0.77–0.99)	N/A
Liu 2017 (a) [23]	Lung cancer	Case-control, cohort	Vit-D intake	22	813,801	Incidence	6	N/A	OR = 0.89 (0.83–0.97)	N/A
Liu 2017 (b) [23]	Lung cancer	Case-control, cohort	Serum 25(OH)D	22	813,801	Incidence, mortality	8	3	OR = 0.72 (0.61–0.85)	OR = 0.39 (0.28–0.54)
Liu 2018 (a) * [47]	Pancreatic cancer	Case-control, cohort, RCTs	Vit-D intake	25	1,213,821	Incidence	11	N/A	RR = 0.90 (0.83–0.98)	N/A
Liu 2018 (b) ** [47]	Pancreatic cancer	Case-control, cohort, RCTs	Vit-D intake	25	1,213,821	Incidence	14	N/A	RR = 0.79 (0.73–0.85)	N/A
Lopez-Caleya 2022 [48]	Colorectal cancer	Case-control	Vit-D intake	55	55,522	Incidence	23	N/A	OR = 0.96 (0.93–0.98)	N/A
Maalmi 2014 *** [49]	Breast cancer	Cohort	Serum 25(OH)D	5	4413	Mortality	N/A	3	N/A	HR = 0.57 (0.38–0.84)
Maalmi 2018 *** [50]	Colorectal cancer	Cohort	Serum 25(OH)D	11	7718	Mortality	N/A	6	N/A	HR = 0.67 (0.57–0.78)
Pu 2021 (a) [51]	Head and neck cancer	Case-control, cohort	Vit-D intake	16	81,908	Incidence	3	N/A	OR = 0.77 (0.65–0.92)	N/A
Pu 2021 (b) [51]	Head and neck cancer	Case-control, cohort	Serum 25(OH)D	16	81,908	Incidence, mortality	5	3	OR = 0.68 (0.59–0.78)	OR = 0.75 (0.60–0.94)
Shahvazi 2019 [52]	Prostate cancer	Clinical trials	Vit-D intake	22	1902	Mortality	N/A	3	N/A	RR = 1.05 (0.81–1.36)
Sun 2021 (a) [24]	Lung cancer	Case-control, cohort, RCTs	Serum 25(OH)D	40	1,566,662	Incidence, mortality	16	9	RR = 0.91 (0.84–0.98)	RR = 0.71 (0.53–0.97)
Sun 2021 (b) [24]	Lung cancer	Case-control, cohort, RCTs	Vit-D intake	40	1,566,662	Incidence	4	N/A	RR = 0.90 (0.80–1.03)	N/A
Wei 2018 (a) [53]	Lung cancer	Case-control, cohort	Serum 25(OH)D	16	280,127	Incidence	12	N/A	RR = 1.04 (0.94–1.15)	N/A
Wei 2018 (b) [53]	Lung cancer	Case-control, cohort	Vit-D intake	16	280,127	Incidence	5	N/A	RR = 0.85 (0.74–0.98)	N/A
Xu 2021 [54]	Colorectal cancer	Case-control, cohort	Vit-D intake	25	911,638	Incidence	21	N/A	OR = 0.87 (0.82–0.92)	N/A
Zhang 2017 (a) [55]	Pancreatic cancer	Case-control, cohort	Serum 25(OH)D	12	893,168	Incidence, mortality	5	5	RR = 1.02 (0.66–1.57)	HR = 0.81 (0.68–0.96)
Zhang 2017 (b) [55]	Pancreatic cancer	Case-control, cohort	Vit-D intake	12	893,168	Incidence	2	N/A	RR = 1.11 (0.67–1.86)	N/A
Zhang 2019 [56]	Total cancer	RCTs	Vit-D intake	10	81,362	Incidence, mortality	10	7	RR = 0.99 (0.94–1.03)	RR = 0.87 (0.79–0.95)
Zhang 2021 [57]	Liver cancer	Cohort	Serum 25(OH)D	6	6357	Incidence	6	N/A	HR = 0.53 (0.41–0.68)	N/A
Zhang 2022 [58]	Total cancer	RCTs	Vit-D intake	12	72,669	Incidence, mortality	11	6	RR = 0.99 (0.93–1.06)	RR = 0.96 (0.80–1.16)
Zhao 2016 [59]	Bladder cancer	Case-control, cohort	Serum 25(OH)D	7	90,757	Incidence	7	N/A	OR = 0.76 (0.66–1.87)	N/A
Zhou 2020 [60]	Breast cancer	RCTs	Vit-D intake	8	72,275	Incidence	6	N/A	RR = 1.04 (0.85–1.29)	N/A

RCTs randomized controlled trials, HR hazard ratio, NOP number of studies, OR odds ratio, CI confidence interval, Vit-D Vitamin D, N/R not reported, RR risk ratio, N/A not available or data missing, ^x^ case-control studies, ^y^ prospective cohort studies, 25(OH)D 25-hidroksivitamin-D, * prospective studies, ** retrospective studies, *** cancer related mortality.

**Table 2:** Subgroup analyses examining specific characteristics and factors related to vitamin D in studies incorporated in the meta-meta-analysis.

Analysis	Model	Number of Reports (n)	Effect Size (ES) (OR or RR)	95% CI	*p* Value	*I* ^2^	*p* Value	Intercept	Tau (t)	*p* Value
Vitamin D intake and cancer risk *										
Total cancer	Fixed	7	0.99 **	0.97–1.01	0.300	0.00	0.983	0.37	0.72	0.506
Colorectal carcinoma	Random	6	0.89 **	0.83–0.96	0.002	79.4	<0.001	−2.11	−1.70	0.164
Lung cancer	Fixed	3	0.88 **	0.83–0.94	<0.001	0.00	0.817	−0.72	−0.59	0.658
RCTs ***	Fixed	8	0.99 **	0.97–1.01	0.320	0.00	0.988	0.49	1.35	0.227
Observational	Random	14	0.90 **	0.86–0.95	<0.001	68.43	<0.001	−1.09	−1.51	0.156
Serum 25 (OH)D levels and cancer risk *										
Colorectal carcinoma	Fixed	4	0.65 **	0.60–0.70	<0.001	48.4	0.121	3.23	1.21	0.351
Lung cancer	Random	3	0.89 **	0.75–1.05	0.178	85.84	0.001	−4.32	−0.68	0.619
Vitamin D intake and cancer related mortality *										
Total cancer	Fixed	7	0.89 ****	0.85–0.93	<0.001	0.00	0.929	0.77	0.98	0.372
RCTs ***	Fixed	7	0.89 ****	0.85–0.93	<0.001	0.00	0.929	0.77	0.98	0.372

OR odds ratio, ES effect size, RR risk ratio, N/R not reported, RCTs randomized controlled trials, N/A not available or missing data, CI confidence interval, * Meta-meta-analysis was not conducted for types of cancer with fewer than three reports ** OR, *** studies containing only RCTs were included, studies with a combination of other study types and RCTs were excluded, **** RR.

## Data Availability

To obtain the datasets, interested parties should contact the corresponding author (MEA) directly and submit a reasonable request for access to the data. The corresponding author (MEA) will provide further information and guidance on accessing the datasets.

## Supplementary Materials

https://www.mdpi.com/article/10.3390/nu15122722/s1, Table S1: The PRISMA checklist; Table S2: Search strategy developed for meta-meta analysis in Pubmed, Web of Science and Scopus databases; Table S3: AMSTAR-2 checklist; Table S4: Summary of Phase 2 domains, Phase 3, and signaling questions of Risk of Bias in Systematic reviews (ROBIS); Table S5: Results of ROBIS assessments; Table S6: List of included studies; Table S7: List of excluded studies; Figure S1: Sensitivity analysis associated with vitamin D intake (low and high intake) and cancer risk; Figure S2: Sensitivity analysis associated with serum 25(OH)D levels (low and high levels) and cancer risk; Figure S3: Bubble chart showing the publication years of Vitamin D-related studies, the number of studies included, and sample sizes (bubble size indicates sample size).

## Abbreviations

HR: Hazard ratio; GLOBOCAN: Global Cancer Observatory; OR: Odds ratio; WHO: World Health Organization; Vit-D: Vitamin-D; QoL: Quality of life; 25(OH)D: 25-hydroxyvitamin-D; MeSH: Medical Subject Headings; WoS: Web of Science; RR: Risk ratio.
