# Supplementary material for: Vitamin D Intake, Serum 25-Hydroxyvitamin-D (25(OH)D) Levels, and Cancer Risk: A Comprehensive Meta-Meta-Analysis Including Meta-Analyses of Randomized Controlled Trials and Observational Epidemiological Studies
Source: Nutrients. 2023 Jun 12;15(12):2722. doi: 10.3390/nu15122722 (PMC10302100; doi:10.3390/nu15122722)
Supplement: Supplementary file 1 [file nutrients-15-02722-s001.zip › nutrients-2408754-supplementary.pdf]

Supplementary appendix

Table S1 The PRISMA checklist

Section/topic	#	Checklist item	Reported on page #
**TITLE**			
Title	1	Identify the report as a systematic review, meta-analysis, or both.	1
**ABSTRACT**			
Structured summary	2	Provide a structured summary including, as applicable: background; objectives; data sources; study eligibility criteria, participants, and interventions; study appraisal and synthesis methods; results; limitations; conclusions and implications of key findings; systematic review registration number.	2
**INTRODUCTION**			
Rationale	3	Describe the rationale for the review in the context of what is already known.	4-5
Objectives	4	Provide an explicit statement of questions being addressed with reference to participants, interventions, comparisons, outcomes, and study design (PICOS).	4-5
**METHODS**			
Protocol and registration	5	Indicate if a review protocol exists, if and where it can be accessed (e.g., Web address), and, if available, provide registration information including registration number.	5-8
Eligibility criteria	6	Specify study characteristics (e.g., PICOS, length of follow-up) and report characteristics (e.g., years considered, language, publication status) used as criteria for eligibility, giving rationale.	5-8
Information sources	7	Describe all information sources (e.g., databases with dates of coverage, contact with study authors to identify additional studies) in the search and date last searched.	5-6
Search	8	Present full electronic search strategy for at least one database, including any limits used, such that it could be repeated.	5-8
Study selection	9	State the process for selecting studies (i.e., screening, eligibility, included in systematic review, and, if applicable, included in the meta-analysis).	5-8
Data collection process	10	Describe method of data extraction from reports (e.g., piloted forms, independently, in duplicate) and any processes for obtaining and confirming data from investigators.	5-6
Data items	11	List and define all variables for which data were sought (e.g., PICOS, funding sources) and any assumptions and simplifications made.	5-7
Risk of bias in individual studies	12	Describe methods used for assessing risk of bias of individual studies (including specification of whether this was done at the study or outcome level), and how this information is to be used in any data synthesis.	5-7
Summary measures	13	State the principal summary measures (e.g., risk ratio, difference in means).	6-8

Section/topic	#	Checklist item	Reported on page #
Synthesis of results	14	Describe the methods of handling data and combining results of studies, if done, including measures of consistency (e.g., I^2) for each meta-analysis.	6-8
**Section/topic**	**#**	**Checklist item**	**Reported on page #**
Risk of bias across studies	15	Specify any assessment of risk of bias that may affect the cumulative evidence (e.g., publication bias, selective reporting within studies).	7-8
Additional analyses	16	Describe methods of additional analyses (e.g., sensitivity or subgroup analyses, meta-regression), if done, indicating which were pre-specified.	7-8
**RESULTS**			
Study selection	17	Give numbers of studies screened, assessed for eligibility, and included in the review, with reasons for exclusions at each stage, ideally with a flow diagram.	8
Study characteristics	18	For each study, present characteristics for which data were extracted (e.g., study size, PICOS, follow-up period) and provide the citations.	8-9
Risk of bias within studies	19	Present data on risk of bias of each study and, if available, any outcome level assessment (see item 12).	8-9
Results of individual studies	20	For all outcomes considered (benefits or harms), present, for each study: (a) simple summary data for each intervention group (b) effect estimates and confidence intervals, ideally with a forest plot.	8-9
Synthesis of results	21	Present results of each meta-analysis done, including confidence intervals and measures of consistency.	9-12
Risk of bias across studies	22	Present results of any assessment of risk of bias across studies (see Item 15).	9-12
Additional analysis	23	Give results of additional analyses, if done (e.g., sensitivity or subgroup analyses, meta-regression [see Item 16]).	11-12
**DISCUSSION**			
Summary of evidence	24	Summarize the main findings including the strength of evidence for each main outcome; consider their relevance to key groups (e.g., healthcare providers, users, and policy makers).	12-14
Limitations	25	Discuss limitations at study and outcome level (e.g., risk of bias), and at review-level (e.g., incomplete retrieval of identified research, reporting bias).	14
Conclusions	26	Provide a general interpretation of the results in the context of other evidence, and implications for future research.	14-15
**FUNDING**			
Funding	27	Describe sources of funding for the systematic review and other support (e.g., supply of data); role of funders for the systematic review.	15

Table S2 Search strategy developed for meta-meta analysis in Pubmed, Web of Science and Scopus databases

**Database**	**Keywords**
**Pubmed/Medline**	
#1	Search: **(((((((Vitamin D[Title/Abstract]) OR (D vitamin[Title/Abstract])) OR (calciferol[Title/Abstract])) OR (cholecalciferol[Title/Abstract])) OR (cholecalciferol-D3[Title/Abstract])) OR (Vitamin-D3[Title/Abstract])) OR (25-hydroxy vitamin D[Title/Abstract])) OR (25-hydroxy vitamin D3[Title/Abstract])** Sort by: **Most Recent**<br>"vitamin d"[Title/Abstract] OR "d vitamin"[Title/Abstract] OR "calciferol"[Title/Abstract] OR "cholecalciferol"[Title/Abstract] OR "cholecalciferol-D3"[Title/Abstract] OR "Vitamin-D3"[Title/Abstract] OR "25 hydroxy vitamin d"[Title/Abstract] OR "25 hydroxy vitamin d3"[Title/Abstract]
#2	"cancer s"[All Fields] OR "cancerated"[All Fields] OR "canceration"[All Fields] OR "cancerization"[All Fields] OR "cancerized"[All Fields] OR "cancerous"[All Fields] OR "neoplasms"[MeSH Terms] OR "neoplasms"[All Fields] OR "cancer"[All Fields] OR "cancers"[All Fields] OR "cysts"[MeSH Terms] OR "cysts"[All Fields] OR "cyst"[All Fields] OR "neurofibroma"[MeSH Terms] OR "neurofibroma"[All Fields] OR "neurofibromas"[All Fields] OR "tumor s"[All Fields] OR "tumoral"[All Fields] OR "tumorous"[All Fields] OR "tumour"[All Fields] OR "neoplasms"[MeSH Terms] OR "neoplasms"[All Fields] OR "tumor"[All Fields] OR "tumour s"[All Fields] OR "tumoural"[All Fields] OR "tumourous"[All Fields] OR "tumours"[All Fields] OR "tumors"[All Fields] OR "neoplasm s"[All Fields] OR "neoplasms"[MeSH Terms] OR "neoplasms"[All Fields] OR "neoplasm"[All Fields] OR "malign"[All Fields] OR "malignance"[All Fields] OR "malignances"[All Fields] OR "malignant"[All Fields] OR "malignants"[All Fields] OR "malignities"[All Fields] OR "malignity"[All Fields] OR "malignization"[All Fields] OR "malignized"[All Fields] OR "maligns"[All Fields] OR "neoplasms"[MeSH Terms] OR "neoplasms"[All Fields] OR "malignancies"[All Fields] OR "malignancy"[All Fields]<br>**Translations**<br>**cancer:** "cancer's"[All Fields] OR "cancerated"[All Fields] OR "canceration"[All Fields] OR "cancerization"[All Fields] OR "cancerized"[All Fields] OR "cancerous"[All Fields] OR "neoplasms"[MeSH Terms] OR "neoplasms"[All Fields] OR "cancer"[All Fields] OR "cancers"[All Fields]<br>**tumour:** "cysts"[MeSH Terms] OR "cysts"[All Fields] OR "cyst"[All Fields] OR "neurofibroma"[MeSH Terms] OR "neurofibroma"[All Fields] OR "neurofibromas"[All Fields] OR "tumor's"[All Fields] OR "tumoral"[All Fields] OR "tumorous"[All Fields] OR "tumour"[All Fields] OR "neoplasms"[MeSH Terms] OR "neoplasms"[All Fields] OR "tumor"[All Fields] OR "tumour's"[All Fields] OR "tumoural"[All

	Fields] OR "tumourous"[All Fields] OR "tumours"[All Fields] OR "tumors"[All Fields]<br>**neoplasms:** "neoplasm's"[All Fields] OR "neoplasms"[MeSH Terms] OR "neoplasms"[All Fields] OR "neoplasm"[All Fields]<br>**malignancy:** "malign"[All Fields] OR "malignance"[All Fields] OR "malignances"[All Fields] OR "malignant"[All Fields] OR "malignants"[All Fields] OR "malignities"[All Fields] OR "malignity"[All Fields] OR "malignization"[All Fields] OR "malignized"[All Fields] OR "maligns"[All Fields] OR "neoplasms"[MeSH Terms] OR "neoplasms"[All Fields] OR "malignancies"[All Fields] OR "malignancy"[All Fields]
#3	Search: **((meta-analysis[Title/Abstract]) OR (systematic review with meta-analysis[Title/Abstract])) OR (systematic review[Title/Abstract] AND meta-analysis[Title/Abstract])** Sort by: **Most Recent**<br>"meta-analysis"[Title/Abstract] OR "systematic review with meta analysis"[Title/Abstract] OR ("systematic review"[Title/Abstract] AND "meta-analysis"[Title/Abstract])
#4	#1 AND #2 AND #3<br><br>Search: **((((((((Vitamin D[Title/Abstract]) OR (D vitamin[Title/Abstract])) OR (calciferol[Title/Abstract])) OR (cholecalciferol[Title/Abstract])) OR (cholecalciferol-D3[Title/Abstract])) OR (Vitamin-D3[Title/Abstract])) OR (25-hydroxy vitamin D[Title/Abstract])) OR (25-hydroxy vitamin D3[Title/Abstract])) AND ("cancer s"[All Fields] OR "cancerated"[All Fields] OR "canceration"[All Fields] OR "cancerization"[All Fields] OR "cancerized"[All Fields] OR "cancerous"[All Fields] OR "neoplasms"[MeSH Terms] OR "neoplasms"[All Fields] OR "cancer"[All Fields] OR "cancers"[All Fields] OR "cysts"[MeSH Terms] OR "cysts"[All Fields] OR "cyst"[All Fields] OR "neurofibroma"[MeSH Terms] OR "neurofibroma"[All Fields] OR "neurofibromas"[All Fields] OR "tumor s"[All Fields] OR "tumoral"[All Fields] OR "tumorous"[All Fields] OR "tumour"[All Fields] OR "neoplasms"[MeSH Terms] OR "neoplasms"[All Fields] OR "tumor"[All Fields] OR "tumour s"[All Fields] OR "tumoural"[All Fields] OR "tumourous"[All Fields] OR "tumours"[All Fields] OR "tumors"[All Fields] OR "neoplasm s"[All Fields] OR "neoplasms"[MeSH Terms] OR "neoplasms"[All Fields] OR "neoplasm"[All Fields] OR "malign"[All Fields] OR "malignance"[All Fields] OR "malignances"[All Fields] OR "malignant"[All Fields] OR "malignants"[All Fields] OR "malignities"[All Fields] OR "malignity"[All Fields] OR "malignization"[All Fields] OR "malignized"[All Fields] OR "maligns"[All Fields] OR "neoplasms"[MeSH Terms] OR "neoplasms"[All Fields] OR "malignancies"[All Fields] OR "malignancy"[All Fields])) AND (((meta-analysis[Title/Abstract]) OR (systematic review with meta-analysis[Title/Abstract])) OR**

	**(systematic review[Title/Abstract] AND meta-analysis[Title/Abstract]))** Sort by: **Most Recent**<br>("vitamin d"[Title/Abstract] OR "d vitamin"[Title/Abstract] OR "calciferol"[Title/Abstract] OR "cholecalciferol"[Title/Abstract] OR "cholecalciferol-D3"[Title/Abstract] OR "Vitamin-D3"[Title/Abstract] OR "25 hydroxy vitamin d"[Title/Abstract] OR "25 hydroxy vitamin d3"[Title/Abstract]) AND ("cancer s"[All Fields] OR "cancerated"[All Fields] OR "canceration"[All Fields] OR "cancerization"[All Fields] OR "cancerized"[All Fields] OR "cancerous"[All Fields] OR "neoplasms"[MeSH Terms] OR "neoplasms"[All Fields] OR "cancer"[All Fields] OR "cancers"[All Fields] OR "cysts"[MeSH Terms] OR "cysts"[All Fields] OR "cyst"[All Fields] OR "neurofibroma"[MeSH Terms] OR "neurofibroma"[All Fields] OR "neurofibromas"[All Fields] OR "tumor s"[All Fields] OR "tumoral"[All Fields] OR "tumorous"[All Fields] OR "tumour"[All Fields] OR "neoplasms"[MeSH Terms] OR "neoplasms"[All Fields] OR "tumor"[All Fields] OR "tumour s"[All Fields] OR "tumoural"[All Fields] OR "tumourous"[All Fields] OR "tumours"[All Fields] OR "tumors"[All Fields] OR "neoplasm s"[All Fields] OR "neoplasms"[MeSH Terms] OR "neoplasms"[All Fields] OR "neoplasm"[All Fields] OR "malign"[All Fields] OR "malignance"[All Fields] OR "malignances"[All Fields] OR "malignant"[All Fields] OR "malignants"[All Fields] OR "malignities"[All Fields] OR "malignity"[All Fields] OR "malignization"[All Fields] OR "malignized"[All Fields] OR "maligns"[All Fields] OR "neoplasms"[MeSH Terms] OR "neoplasms"[All Fields] OR "malignancies"[All Fields] OR "malignancy"[All Fields]) AND ("meta-analysis"[Title/Abstract] OR "systematic review with meta analysis"[Title/Abstract] OR ("systematic review"[Title/Abstract] AND "meta-analysis"[Title/Abstract]))
**Web of Science (WoS)**	
#1	ALL=(Vitamin D OR D vitamin OR calciferol OR cholecalciferol OR cholecalciferol-D3 OR Vitamin-D3 OR 25-hydroxy vitamin D OR 25-hydroxy vitamin D3) Indexes=SCI-EXPANDED, SSCI, A&HCI, CPCI-S, CPCI-SSH, BKCIS, BKCI-SSH, ESCI, CCR-EXPANDED
#2	ALL=(cancer OR tumour OR neoplasms OR malignancy) Indexes=SCI-EXPANDED, SSCI, A&HCI, CPCI-S, CPCI-SSH, BKCIS, BKCI-SSH, ESCI, CCR-EXPANDED
#3	ALL=(meta-analysis OR systematic review with meta-analysis OR systematic review AND meta-analysis) Indexes=SCI-EXPANDED, SSCI, A&HCI, CPCI-S, CPCI-SSH, BKCIS, BKCI-SSH, ESCI, CCR-EXPANDED
#4	#1 AND #2 AND #3 Indexes=SCI-EXPANDED, SSCI, A&HCI, CPCI-S, CPCI-SSH, BKCIS, BKCI-SSH, ESCI, CCR-EXPANDED
**Scopus**	
#1	Cancer
#2	malignan*
#3	tumour
#4	neoplasm

#5	(TITLE-ABS-KEY (cancer)) OR (TITLE-ABSKEY (malignan*)) OR (TITLE-ABSKEY (tumour)) OR (TITLE-ABS-KEY (neoplasm))
#6	Vitamin D
#7	calciferol
#8	cholecalciferol
#9	cholecalciferol-D3
#10	Vitamin-D3
#11	25-hydroxy vitamin D
#12	25-hydroxy vitamin D3
#13	Meta-analysis
#14	#5 AND (#6 OR #7 OR #8 OR #9 OR #10 OR #11 OR #12) AND #13

Table S3 AMSTAR-2 checklist

	Item 1	**Item 2**	Item 3	**Item 4**	Item 5	Item 6	**Item 7**	Item 8	**Item 9**	Item 10	**Item 11**	Item 12	**Item 13**	Item 14	**Item 15**	Item 16
Boughanem 2021 (1)	○	○	○	○	○	○	●	○	●	●	○	●	●	○	○	○
Cheema 2022 (2)	○	○	○	○	○	○	○	○	○	●	○	○	○	○	○	○
Chen 2022 (3)	○	●	○	○	●	○	○	●	●	●	○	●	●	○	○	○
Ekmekcioglu 2017 (4)	○	●	○	○	○	○	●	○	●	●	○	●	●	○	○	○
Gao 2018 (5)	○	●	○	○	○	○	●	○	●	●	○	●	●	○	○	○
Goulão 2018 (6)	○	●	○	○	○	○	●	○	●	●	○	●	●	○	○	○
Guo 2020 (7)	○	●	○	○	○	○	●	○	●	●	○	●	●	○	○	○
Guo 2022 (8)	○	○	○	○	○	○	●	○	○	●	○	○	○	○	○	○
Han 2019 (9)	○	●	○	○	○	○	○	○	●	●	○	●	●	○	○	○
Haykal 2019 (10)	○	●	○	●	○	○	●	○	●	●	○	●	●	○	○	○
Hernandez-Alonso 2023 (11)	○	○	○	○	○	○	●	○	●	●	○	●	●	○	○	○
Huncharek 2009 (12)	○	●	○	○	●	●	●	●	●	●	○	●	●	○	○	○
Keum 2014 (13)	○	●	○	○	○	○	○	○	●	●	○	●	●	○	○	○
Khayatzadeh 2015 (14)	○	●	○	○	○	○	●	○	●	●	○	●	●	○	○	○
Kim 2014 (15)	○	●	○	○	●	●	●	○	●	●	○	●	●	○	○	○
Lee 2011 (16)	○	●	○	●	●	●	●	●	●	●	○	●	●	○	○	○
Liao 2015 (18)	○	●	○	○	●	●	○	○	●	●	○	●	●	○	○	○
Liao 2020 (17)	○	●	○	○	○	○	●	○	●	●	○	●	●	○	○	○
Liu 2015 (21)	○	●	○	○	○	○	○	○	●	●	○	●	●	○	○	○
Liu 2017 (19)	○	●	○	○	○	○	○	○	●	●	○	●	●	○	○	○
Liu 2018 (20)	○	●	○	○	○	○	●	○	●	●	○	●	●	○	○	○
Lopez-Caleya 2022 (22)	○	○	○	○	○	○	●	○	●	●	○	●	●	○	○	○
Maalmi 2014 (23)	○	●	○	●	○	○	○	○	●	●	○	●	●	○	○	○
Maalmi 2018 (24)	○	●	○	○	○	○	○	○	●	●	○	●	●	○	○	○
Pu 2021 (25)	○	○	○	○	○	○	○	○	●	●	○	●	●	○	○	○
Shahvazi 2019 (26)	○	○	○	○	○	○	○	○	●	●	○	●	●	○	○	○
Sun 2021 (27)	○	●	○	○	○	○	●	○	●	●	○	●	●	○	○	○
Wei 2018 (28)	○	●	○	○	○	○	●	○	●	●	○	●	●	○	○	○

Xu 2021 (29)	○	●	○	○	○	○	●	○	●	●	○	●	●	○	○	○
Zhang 2017 (31)	○	●	○	○	○	○	●	○	●	●	○	●	●	○	○	○
Zhang 2019 (32)	○	●	○	○	○	○	●	○	●	●	○	●	●	○	○	○
Zhang 2021 (33)	○	●	○	○	○	○	●	○	●	●	○	●	●	○	○	○
Zhang 2022 (30)	○	○	○	○	○	○	●	○	●	●	○	●	●	○	○	○
Zhao 2016 (34)	○	●	○	○	○	○	●	○	●	●	○	●	●	○	○	○
Zhou 2020 (35)	○	●	○	○	○	○	○	○	●	●	○	●	●	○	○	○

○ = Yes, ● = No

Item 1: “Did the research questions and inclusion criteria for the review include the components of PICO?” **Item 2:** "Did the report of the review contain an explicit statement that the review methods were established prior to the conduct of the review and did the report justify any significant deviations from the protocol?" Item 3: “Did the review authors explain their selection of the study designs for inclusion in the review?” **Item 4:** “Did the review authors use a comprehensive literature search strategy?” Item 5: “Did the review authors perform study selection in duplicate?” Item 6 - Did the review authors perform data extraction in duplicate?” **Item 7:** “Did the review authors provide a list of excluded studies and justify the exclusions?” Item 8 - Did the review authors describe the included studies in adequate detail?” **Item 9:** "Did the review authors use a satisfactory technique for assessing the risk of bias (RoB) in individual studies that were included in the review?" Item 10: “Did the review authors report on the sources of funding for the studies included in the review?” **Item 11:** "If meta-analysis was performed did the review authors use appropriate methods for statistical combination of results?" Item 12: "If meta-analysis was performed, did the review authors assess the potential impact of RoB in individual studies on the results of the meta-analysis or other evidence synthesis?" **Item 13:** "Did the review authors account for RoB in individual studies when interpreting/ discussing the results of the review?" Item 14: "Did the review authors provide a satisfactory explanation for, and discussion of, any heterogeneity observed in the results of the review?” **Item 15:** "If they performed quantitative synthesis did the review authors carry out an adequate investigation of publication bias (small study bias) and discuss its likely impact on the results of the review?" Item 16: "Did the review authors report any potential sources of conflict of interest, including any funding they received for conducting the review?"

Table S4 Summary of Phase 2 domains, Phase 3, and signaling questions of Risk of Bias in Systematic reviews (ROBIS)

	Phase 2				Phase 3
	**1. Study eligibility criteria**	**2. Identification and selection of studies**	**3. Data collection and study appraisal**	**4. Synthesis and findings**	**Risk of bias in the review**
Signaling questions	1.1 Did the review adhere to predefined objectives and eligibility criteria?	2.1 Did the search include an appropriate range of databases/ electronic sources for published and unpublished reports?	3.1. Were efforts made to minimize error in data collection?	4.1. Did the synthesis include all studies that it should?	A. Did the interpretation of findings address all of the concerns identified in domains 1 to 4?
	1.2 Were the eligibility criteria appropriate for the review question?	2.2 Were methods additional to database searching used to identify relevant reports?	3.2. Were sufficient study characteristics available for both review authors and readers to be able to interpret the results?	4.2. Were all predefined analyses reported or departures explained?	B. Was the relevance of identified studies to the review's research question appropriately considered?
	1.3 Were eligibility criteria unambiguous?	2.3 Were the terms and structure of the search strategy likely to retrieve as many eligible studies as possible?	3.3. Were all relevant study results collected for use in the synthesis?	4.3. Was the synthesis appropriate given the nature and similarity in the research questions, study designs, and outcomes across included studies?	C. Did the reviewers avoid emphasizing results on the basis of their statistical significance?
	1.4 Were all restrictions in eligibility criteria based on study characteristics appropriate?	2.4 Were restrictions based on date, publication format, or language appropriate?	3.4. Was risk of bias (or methodologic quality) formally assessed using appropriate criteria?	4.4. Was between-study variation minimal or addressed in the synthesis?	
	1.5 Were any restrictions in eligibility criteria based on sources of information appropriate?	2.5 Were efforts made to minimize error in selection of studies?	3.5. Were efforts made to minimize error in risk of bias assessment?	4.5. Were the findings robust, for example, as demonstrated through funnel plot or sensitivity analyses?	
				4.6. Were biases in primary studies minimal or addressed in the synthesis?	
Judgment	Concerns regarding specification of study eligibility criteria	Concerns regarding methods used to identify and/or select studies	Concerns regarding methods used to collect data and appraise studies	Concerns regarding the synthesis	Risk of bias in the review

Table S5 Results of ROBIS assessments

Study	Phase 1	Phase 2				Phase 3
	Assessing relevance	Study eligibility criteria (Domain 1)	Identification and selection of studies (Domain 2)	Data collection and study appraisal (Domain 3)	Synthesis and findings (Domain 4)	Risk of bias in the review
Boughanem 2021 (1)	☺	☺	☹	☺	☺	☺
Cheema 2022 (2)	☺	☺	☺	☺	☺	☺
Chen 2022 (3)	☺	☺	☹	☺	☺	☺
Ekmekcioglu 2017 (4)	☺	☺	☹	☺	☺	☺
Gao 2018 (5)	☺	☺	☺	☹	☺	☺
Goulão 2018 (6)	☺	☺	☹	☺	☺	☺
Guo 2020 (7)	☺	☺	☺	☹	☺	☺
Guo 2022 (8)	☺	☺	☺	☺	☺	☺
Han 2019 (9)	☺	☺	☺	☹	☺	☺

Haykal 2019 (10)	☺	☺	☺	☹	☺	☺
Hernandez-Alonso 2023 (11)	☺	☺	☺	☺	☹	☺
Huncharek 2009 (12)	☺	☺	☹	☺	☹	☹
Keum 2014 (13)	☺	☺	☺	☺	☺	☺
Khayatzadeh 2015 (14)	☺	☺	☺	☹	☺	☺
Kim 2014 (15)	☺	☺	☹	☺	☺	☺
Lee 2011 (16)	☺	☺	☹	☺	☹	☹
Liao 2015 (18)	☺	☺	☺	☺	☹	☺
Liao 2020 (17)	☺	☺	☺	☺	☹	☺
Liu 2015 (21)	☺	☺	☹	☺	☺	☺
Liu 2017 (19)	☺	☺	☺	☹	☺	☺
Liu 2018 (20)	☺	☺	☺	☺	☺	☺
Lopez-Caleya 2022 (22)	☺	☺	☹	☺	☹	☹

Maalmi 2014 (23)	☺	☺	☹	☺	☹	☹
Maalmi 2018 (24)	☺	☺	☺	☹	☺	☺
Pu 2021 (25)	☺	☺	☹	☺	☺	☺
Shahvazi 2019 (26)	☺	☺	☹	☹	☺	☹
Sun 2021 (27)	☺	☺	☺	☺	☺	☺
Wei 2018 (28)	☺	☺	☹	☺	☹	☹
Xu 2021 (29)	☺	☺	☹	☺	☺	☺
Zhang 2017 (31)	☺	☺	☺	☹	☺	☺
Zhang 2019 (32)	☺	☺	☺	☺	☺	☺
Zhang 2021 (33)	☺	☺	☹	☺	☹	☹
Zhang 2022 (30)	☺	☺	☺	☺	☺	☺
Zhao 2016 (34)	☺	☺	☹	☹	☺	☹
Zhou 2020 (35)	☺	☺	☺	☺	☹	☺

Table S6 List of included studies

Nr.	Author of main publication	Reference(s)	Reasons for inclusion
**1**	Boughanem 2021 (1)	Boughanem H, Canudas S, Hernandez-Alonso P, Becerra-Tomás N, Babio N, Salas-Salvadó J, et al. Vitamin D Intake and the Risk of Colorectal Cancer: An Updated Meta-Analysis and Systematic Review of Case-Control and Prospective Cohort Studies. Cancers (Basel). 2021;13(11):2814. https://doi.org/10.3390/cancers13112814	Incidence and/or mortality-related RR, OR, and/or HR reported
**2**	Cheema 2022 (2)	Cheema HA, Fatima M, Shahid A, Bouaddi O, Elgenidy A, Rehman AU, et al. Vitamin D supplementation for the prevention of total cancer incidence and mortality: An updated systematic review and meta-analysis. Heliyon. 2022;8(11):e11290. https://doi.org/10.1016/j.heliyon.2022.e11290	Incidence and/or mortality-related RR, OR, and/or HR reported
**3**	Chen 2022 (3)	Chen X, Li L, Liang Y, Huang T, Zhang H, Fan S, Sun W, Wang Y. Relationship of vitamin D intake, serum 25(OH) D, and solar ultraviolet-B radiation with the risk of gastric cancer: A meta-analysis. J Cancer Res Ther. 2022;18(5):1417-1424. https://doi.org/10.4103/jcrt.jcrt_527_21	Incidence and/or mortality-related RR, OR, and/or HR reported
**4**	Ekmekcioglu 2017 (4)	Ekmekcioglu C, Haluza D, Kundi M. 25-Hydroxyvitamin D Status and Risk for Colorectal Cancer and Type 2 Diabetes Mellitus: A Systematic Review and Meta-Analysis of Epidemiological Studies. Int J Environ Res Public Health. 2017;14(2):127. https://doi.org/10.3390/ijerph14020127	Incidence and/or mortality-related RR, OR, and/or HR reported
**5**	Gao 2018 (5)	Gao J, Wei W, Wang G, Zhou H, Fu Y, Liu N. Circulating vitamin D concentration and risk of prostate cancer: A dose-response meta-analysis of prospective studies. Ther Clin Risk Manag. 2018;14:95-104. https://doi.org/10.2147/TCRM.S149325	Incidence and/or mortality-related RR, OR, and/or HR reported
**6**	Goulão 2018 (6)	Goulão B, Stewart F, Ford JA, MacLennan G, Avenell A. Cancer and vitamin D supplementation: a systematic review and meta-analysis. Am J Clin Nutr. 2018;107(4):652-663. https://doi.org/doi:10.1093/ajcn/nqx047	Incidence and/or mortality-related RR, OR, and/or HR reported

**7**	Guo 2020 (7)	Guo XF, Zhao T, Han JM, Li S, Li D. Vitamin D and liver cancer risk: A meta-analysis of prospective studies. Asia Pac J Clin Nutr. 2020;29(1):175-182. https://doi.org/10.6133/apjcn.202003_29(1).0023	Incidence and/or mortality-related RR, OR, and/or HR reported
**8**	Guo 2022 (8)	Guo Z, Huang M, Fan D, Hong Y, Zhao M, Ding R, et al. Association between vitamin D supplementation and cancer incidence and mortality: A trial sequential meta-analysis of randomized controlled trials. Crit Rev Food Sci Nutr. 2022:1-15. https://doi.org/10.1080/10408398.2022.2056574	Incidence and/or mortality-related RR, OR, and/or HR reported
**9**	Han 2019 (9)	Han J, Guo X, Yu X, Liu S, Cui X, Zhang B, et al. 25-Hydroxyvitamin D and Total Cancer Incidence and Mortality: A meta-analysis of prospective cohort studies. Nutrients. 2019;11(10):1-17. https://doi.org/10.3390/nu11102295	Incidence and/or mortality-related RR, OR, and/or HR reported
**10**	Haykal 2019 (10)	Haykal T, Samji V, Zayed Y, Gakhal I, Dhillon H, Kheiri B, et al. The role of vitamin D supplementation for primary prevention of cancer: meta-analysis of randomized controlled trials. J Community Hosp Intern Med Perspect. 2019;9(6):480-488. https:///doi.org/10.1080/20009666.2019.1701839	Incidence and/or mortality-related RR, OR, and/or HR reported
**11**	Hernandez-Alonso 2023 (11)	Hernández-Alonso P, Boughanem H, Canudas S, Becerra-Tomás N, Fernández de la Puente M, Babio N, et al. Circulating vitamin D levels and colorectal cancer risk: A meta-analysis and systematic review of case-control and prospective cohort studies. Crit Rev Food Sci Nutr. 2023;63(1):1-17. https://doi.org/10.1080/10408398.2021.1939649	Incidence and/or mortality-related RR, OR, and/or HR reported
**12**	Huncharek 2009 (12)	Huncharek M, Muscat J, Kupelnick B. Colorectal cancer risk and dietary intake of calcium, vitamin D, and dairy products: A meta-analysis of 26,335 cases from 60 observational studies. Nutr Cancer. 2009;61(1):47-69. https://doi.org/10.1080/01635580802395733	Incidence and/or mortality-related RR, OR, and/or HR reported
**13**	Keum 2014 (13)	Keum N, Giovannucci E. Vitamin D supplements and cancer incidence and mortality: A meta-analysis. Br J Cancer. 2014;111(5):976-80. https://doi.org/10.1038/bjc.2014.294	Incidence and/or mortality-related RR, OR, and/or HR reported

**14**	Khayatzadeh 2015 (14)	Khayatzadeh S, Feizi A, Saneei P, Esmaillzadeh A. Vitamin D intake, serum Vitamin D levels, and risk of gastric cancer: A systematic review and meta-analysis. J Res Med Sci. 2015;20(8):790-6. https://doi.org/10.4103/1735-1995.168404	Incidence and/or mortality-related RR, OR, and/or HR reported
**15**	Kim 2014 (15)	Kim Y, Je Y. Vitamin D intake, blood 25(OH)D levels, and breast cancer risk or mortality: A meta-analysis. Br J Cancer. 2014;110(11):2772-84. https://doi.org/10.1038/bjc.2014.175	Incidence and/or mortality-related RR, OR, and/or HR reported
**16**	Lee 2011 (16)	Lee JE, Li H, Chan AT, Hollis BW, Lee IM, Stampfer MJ, et al. Circulating levels of vitamin D and colon and rectal cancer: the Physicians' Health Study and a meta-analysis of prospective studies. Cancer Prev Res (Phila). 2011;4(5):735-43. https://doi.org10.1158/1940-6207.CAPR-10-0289	Incidence and/or mortality-related RR, OR, and/or HR reported
**17**	Liao 2015 (18)	Liao Y, Huang JL, Qiu MX, Ma ZW. Impact of serum vitamin D level on risk of bladder cancer: a systemic review and meta-analysis. Tumour Biol. 2015;36(3):1567-72. https://doi.org/10.1007/s13277-014-2728-9	Incidence and/or mortality-related RR, OR, and/or HR reported
**18**	Liao 2020 (17)	Liao MQ, Gao XP, Yu XX, Zeng YF, Li SN, Naicker N, et al. Effects of dairy products, calcium and vitamin D on ovarian cancer risk: a meta-analysis of twenty-nine epidemiological studies. Br J Nutr. 2020;124(10):1001-1012. https://doi.org/10.1017/S0007114520001075	Incidence and/or mortality-related RR, OR, and/or HR reported
**19**	Liu 2015 (21)	Liu Y, Yu Q, Zhu Z, Zhang J, Chen M, Tang P, et al. Vitamin and multiple-vitamin supplement intake and incidence of colorectal cancer: a meta-analysis of cohort studies. Med Oncol. 2015;32(1):1-10. https://doi.org/10.1007/s12032-014-0434-5	Incidence and/or mortality-related RR, OR, and/or HR reported
**20**	Liu 2017 (19)	Liu J, Dong Y, Lu C, Wang Y, Peng L, Jiang M, et al. Meta-analysis of the correlation between vitamin D and lung cancer risk and outcomes. Oncotarget. 2017;8(46):81040-81051. https://doi.org/10.18632/oncotarget.18766	Incidence and/or mortality-related RR, OR, and/or HR reported

**21**	Liu 2018 (20)	Liu Y, Wang X, Sun X, Lu S, Liu S. Vitamin intake and pancreatic cancer risk reduction: A meta-analysis of observational studies. Medicine (Baltimore). 2018;97(13):e0114. https://doi.org/10.1097/MD.0000000000010114	Incidence and/or mortality-related RR, OR, and/or HR reported
**22**	Lopez-Caleya 2022 (22)	Lopez-Caleya JF, Ortega-Valín L, Fernández-Villa T, Delgado-Rodríguez M, Martín-Sánchez V, Molina AJ. The role of calcium and vitamin D dietary intake on risk of colorectal cancer: systematic review and meta-analysis of case-control studies. Cancer Causes Control. 2022;33(2):167-182. https://doi.org/10.1007/s10552-021-01512-3	Incidence and/or mortality-related RR, OR, and/or HR reported
**23**	Maalmi 2014 (23)	Maalmi H, Ordóñez-Mena JM, Schöttker B, Brenner H. Serum 25-hydroxyvitamin D levels and survival in colorectal and breast cancer patients: Systematic review and meta-analysis of prospective cohort studies. Eur J Cancer. 2014;50(8):1510-21. https://doi.org/10.1016/j.ejca.2014.02.006	Incidence and/or mortality-related RR, OR, and/or HR reported
**24**	Maalmi 2018 (24)	Maalmi H, Walter V, Jansen L, Boakye D, Schöttker B, Hoffmeister M, et al. Association between blood 25-hydroxyvitamin D levels and survival in colorectal cancer patients: An updated systematic review and meta-analysis. Nutrients. 2018;10(7). https://doi.org/10.3390/nu10070896	Incidence and/or mortality-related RR, OR, and/or HR reported
**25**	Pu 2021 (25)	Pu Y, Zhu G, Xu Y, Zheng S, Tang B, Huang H, et al. Association Between Vitamin D Exposure and Head and Neck Cancer: A Systematic Review With Meta-Analysis. Front Immunol. 2021;12(February):1-11. https://doi.org/10.3389/fimmu.2021.627226	Incidence and/or mortality-related RR, OR, and/or HR reported
**26**	Shahvazi 2019 (26)	Shahvazi S, Soltani S, Ahmadi SM, de Souza RJ, Salehi-Abargouei A. The Effect of Vitamin D Supplementation on Prostate Cancer: A Systematic Review and Meta-Analysis of Clinical Trials. Horm Metab Res. 2019;51(1):11-21. https://doi.org/10.1055/a-0774-8809	Incidence and/or mortality-related RR, OR, and/or HR reported
**27**	Sun 2021 (27)	Sun K, Zuo M, Zhang Q, Wang K, Huang D, Zhang H. Anti-Tumor Effect of Vitamin D Combined with Calcium on Lung Cancer: A Systematic Review and Meta-Analysis. Nutr Cancer. 2021;73(11-12):2633-2642. https://doi.org/10.1080/01635581.2020.1850812	Incidence and/or mortality-related RR, OR, and/or HR reported

**28**	Wei 2018 (28)	Wei H, Jing H, Wei Q, Wei G, Heng Z. Associations of the risk of lung cancer with serum 25-hydroxyvitamin D level and dietary vitamin D intake: A dose-response PRISMA meta-analysis. Medicine (Baltimore). 2018;97(37):e12282. https://doi.org/10.1097/MD.0000000000012282	Incidence and/or mortality-related RR, OR, and/or HR reported
**29**	Xu 2021 (29)	Xu Y, Qian M, Hong J, Ng DM, Yang T, Xu L, Ye X. The effect of vitamin D on the occurrence and development of colorectal cancer: a systematic review and meta-analysis. Int J Colorectal Dis. 2021;36(7):1329-1344. https://doi.org/10.1007/s00384-021-03879-w	Incidence and/or mortality-related RR, OR, and/or HR reported
**30**	Zhang 2017 (31)	Zhang X, Huang XZ, Chen WJ, Wu J, Chen Y, Wu CC, et al. Plasma 25-hydroxyvitamin D levels, vitamin D intake, and pancreatic cancer risk or mortality: A meta-analysis. Oncotarget. 2017;8(38):64395-406. https://doi.org/10.18632/oncotarget.18888	Incidence and/or mortality-related RR, OR, and/or HR reported
**31**	Zhang 2019 (32)	Zhang X, Niu W. Meta-analysis of randomized controlled trials on vitamin D supplement and cancer incidence and mortality. Biosci Rep. 2019;39(11):BSR20190369. https://doi.org/10.1042/BSR20190369	Incidence and/or mortality-related RR, OR, and/or HR reported
**32**	Zhang 2021 (33)	Zhang Y, Jiang X, Li X, Găman MA, Kord-Varkaneh H, Rahmani J, et al. Serum Vitamin D Levels and Risk of Liver Cancer: A Systematic Review and Dose-Response Meta-Analysis of Cohort Studies. Nutr Cancer. 2021;73(8):1-9. https://doi.org/10.1080/01635581.2020.1797127	Incidence and/or mortality-related RR, OR, and/or HR reported
**33**	Zhang 2022 (30)	Zhang R, Zhang Y, Liu Z, Pei Y, Xu P, Chong W, et al. Association between Vitamin D Supplementation and Cancer Mortality: A Systematic Review and Meta-Analysis. Cancers (Basel). 2022;14(15):3717. https://doi.org/10.3390/cancers14153717	Incidence and/or mortality-related RR, OR, and/or HR reported
**34**	Zhao 2016 (34)	Zhao Y, Chen C, Pan W, Gao M, He W, Mao R, et al. Comparative efficacy of vitamin D status in reducing the risk of bladder cancer: A systematic review and network meta-analysis. Nutrition. 2016;32(5):515-23. https://doi.org/10.1016/j.nut.2015.10.023	Incidence and/or mortality-related RR, OR, and/or HR reported

**35**	Zhou 2020 (35)	Zhou L, Chen B, Sheng L, Turner A. The effect of vitamin D supplementation on the risk of breast cancer: a trial sequential meta-analysis. Breast Cancer Res Treat. 2020;182(1):1-8. https://doi.org/10.1007/s10549-020-05669-4	Incidence and/or mortality-related RR, OR, and/or HR reported

HR hazard ratio, *OR* odds ratio, *RR* risk ratio,

Table S7 List of excluded studies

Nr.	Author of main publication	Reference(s)	Reasons for exclusion
**1**	Liu 2022 (36)	Liu X, Zhou Y, Zou X. Correlation between Serum 25-Hydroxyvitamin D Levels and Gastric Cancer: A Systematic Review and Meta-Analysis. Curr Oncol. 2022;29(11):8390-8400. https://doi.org/10.3390/curroncol29110661	Weighted mean difference (WMD) was reported
**2**	Choi 2015 (37)	Choi YJ, Kim YH, Cho CH, Kim SH, Lee JE. Circulating levels of vitamin D and colorectal adenoma: A case-control study and a meta-analysis. World J Gastroenterol. 2015;21(29):8868-77. https://doi.org/10.3748/wjg.v21.i29.8868	Outcome reported for adenoma
**3**	Emmanouilidou 2022 (38)	Emmanouilidou G, Kalopitas G, Bakaloudi DR, Karanika E, Theocharidou E, Germanidis G, et al. Vitamin D as a chemopreventive agent in colorectal neoplasms. A systematic review and meta-analysis of randomized controlled trials. Pharmacol Ther. 2022;237:108252. https://doi.org/10.1016/j.pharmthera.2022.108252	Outcome reported for neoplasm
**4**	Huang 2020 (39)	Huang D, Lei S, Wu Y, Weng M, Zhou Y, Xu J, et al. Additively protective effects of vitamin D and calcium against colorectal adenoma incidence, malignant transformation and progression: A systematic review and meta-analysis. Clin Nutr. 2020;39(8):2525-2538. https://doi.org/10.1016/j.clnu.2019.11.012	Outcome reported for adenoma
**5**	Keum 2022 (40)	Keum N, Chen QY, Lee DH, Manson JE, Giovannucci E. Vitamin D supplementation and total cancer incidence and mortality by daily vs. infrequent large-bolus dosing strategies: a meta-analysis of randomised controlled trials. Br J Cancer. 2022 Sep;127(5):872-878. https://doi.org/10.1038/s41416-022-01850-2	High or regular doses have been reported

**6**	Song 2019 (41)	Song D, Deng Y, Liu K, Zhou L, Li N, Zheng Y, et al. Vitamin D intake, blood vitamin D levels, and the risk of breast cancer: a dose-response meta-analysis of observational studies. Aging (Albany NY). 2019;11(24):12708-12732. https://doi.org/10.18632/aging.102597	The dose-response relationship was examined
**7**	Wei 2008 (42)	Wei MY, Garland CF, Gorham ED, Mohr SB, Giovannucci E. Vitamin D and prevention of colorectal adenoma: a meta-analysis. Cancer Epidemiol Biomarkers Prev. 2008;17(11):2958-69. https://doi.org/10.1158/1055-9965.EPI-08-0402	Outcome reported for adenoma

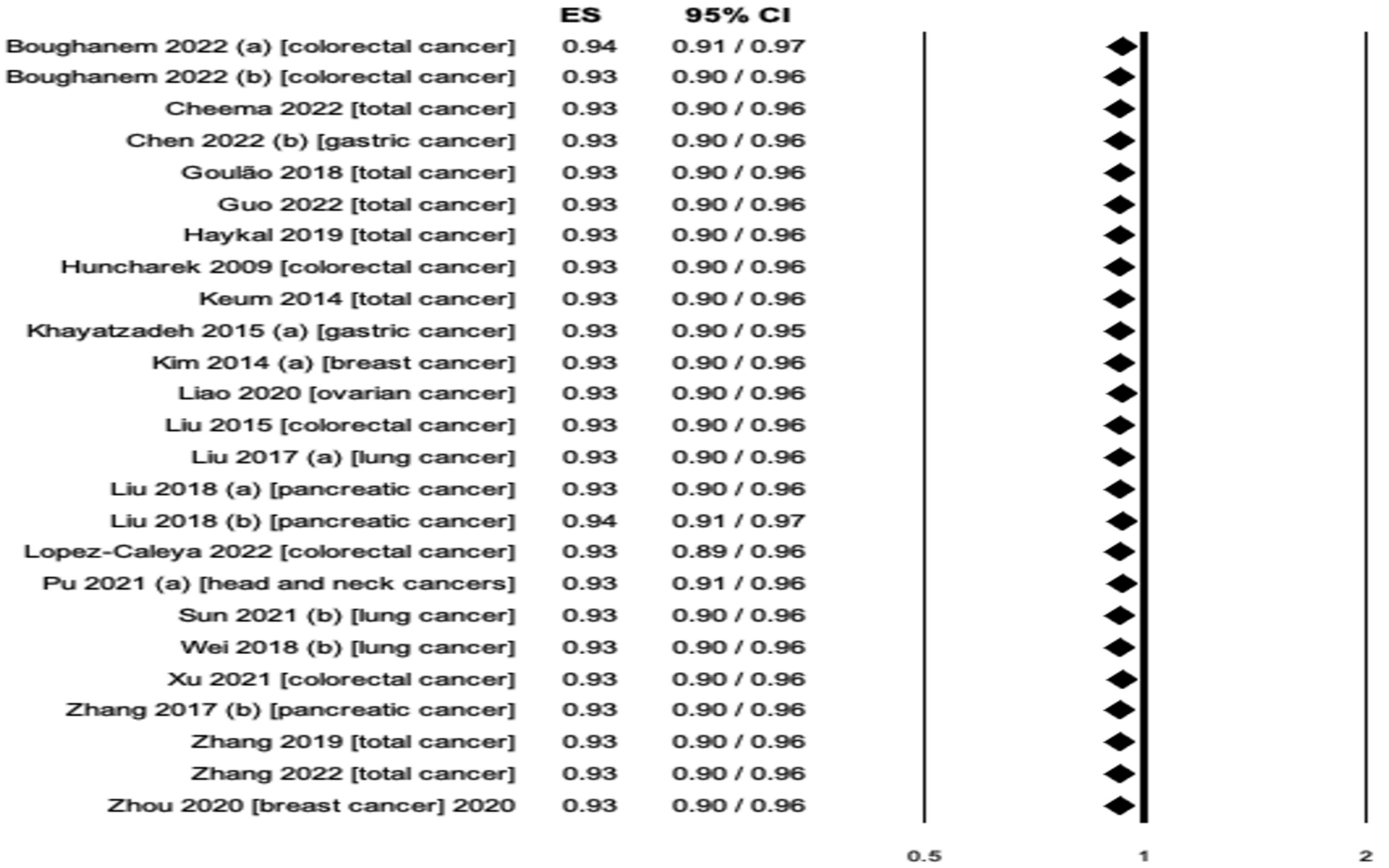

Fig. S1 Sensitivity analysis associated with vitamin D intake (low and high intake) and cancer risk

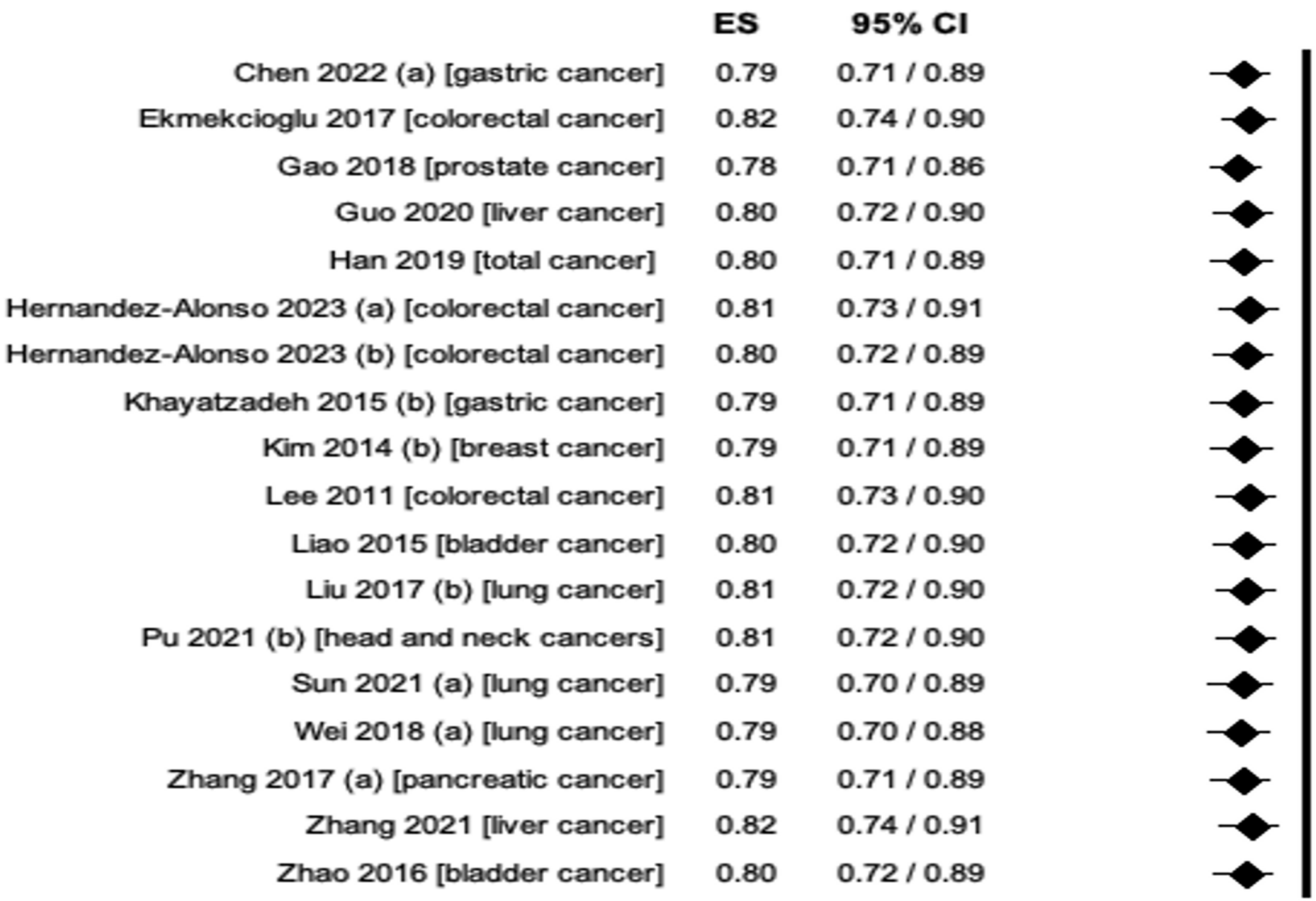

Fig. S2 Sensitivity analysis associated with serum 25(OH)D levels (low and high levels) and cancer risk

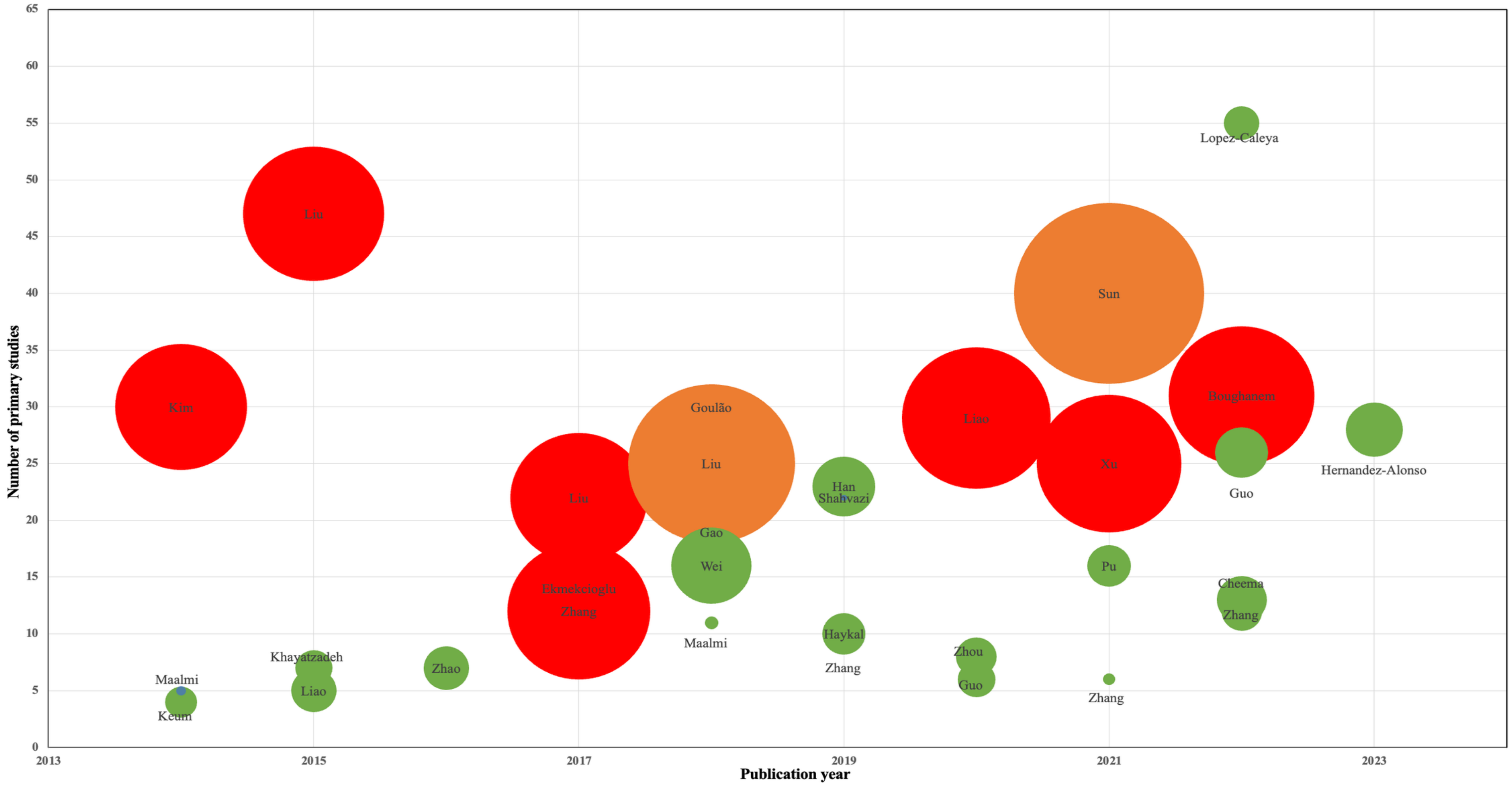

Fig. S3 Bubble chart showing the publication years of Vitamin D-related studies, the number of studies included, and sample sizes (bubble size indicates sample size)

References

1. Boughanem H, Canudas S, Hernandez-Alonso P, Becerra-Tomás N, Babio N, Salas-Salvadó J, et al. Vitamin D Intake and the Risk of Colorectal Cancer: An Updated Meta-Analysis and Systematic Review of Case-Control and Prospective Cohort Studies. Cancers (Basel). 2021;13(11):2814. https://doi.org/10.3390/cancers13112814
2. Cheema HA, Fatima M, Shahid A, Bouaddi O, Elgenidy A, Rehman AU, et al. Vitamin D supplementation for the prevention of total cancer incidence and mortality: An updated systematic review and meta-analysis. Heliyon. 2022;8(11):e11290. https://doi.org/10.1016/j.heliyon.2022.e11290
3. Chen X, Li L, Liang Y, Huang T, Zhang H, Fan S, Sun W, Wang Y. Relationship of vitamin D intake, serum 25(OH) D, and solar ultraviolet-B radiation with the risk of gastric cancer: A meta-analysis. J Cancer Res Ther. 2022;18(5):1417-1424. https://doi.org/10.4103/jcrt.jcrt_527_21
4. Ekmekcioglu C, Haluza D, Kundi M. 25-Hydroxyvitamin D Status and Risk for Colorectal Cancer and Type 2 Diabetes Mellitus: A Systematic Review and Meta-Analysis of Epidemiological Studies. Int J Environ Res Public Health. 2017;14(2):127. https://doi.org/10.3390/ijerph14020127
5. Gao J, Wei W, Wang G, Zhou H, Fu Y, Liu N. Circulating vitamin D concentration and risk of prostate cancer: A dose-response meta-analysis of prospective studies. Ther Clin Risk Manag. 2018;14:95-104. https://doi.org/10.2147/TCRM.S149325
6. Goulão B, Stewart F, Ford JA, MacLennan G, Avenell A. Cancer and vitamin D supplementation: a systematic review and meta-analysis. Am J Clin Nutr. 2018;107(4):652-663. https://doi.org/doi:10.1093/ajcn/nqx047
7. Guo XF, Zhao T, Han JM, Li S, Li D. Vitamin D and liver cancer risk: A meta-analysis of prospective studies. Asia Pac J Clin Nutr. 2020;29(1):175-182. https://doi.org/10.6133/apjcn.202003_29(1).0023
8. Guo Z, Huang M, Fan D, Hong Y, Zhao M, Ding R, et al. Association between vitamin D supplementation and cancer incidence and mortality: A trial sequential meta-analysis of randomized controlled trials. Crit Rev Food Sci Nutr. 2022:1-15. https://doi.org/10.1080/10408398.2022.2056574

9. Han J, Guo X, Yu X, Liu S, Cui X, Zhang B, et al. 25-Hydroxyvitamin D and Total Cancer Incidence and Mortality: A meta-analysis of prospective cohort studies. Nutrients. 2019;11(10):1-17. https://doi.org/10.3390/nu11102295
10. Haykal T, Samji V, Zayed Y, Gakhal I, Dhillon H, Kheiri B, et al. The role of vitamin D supplementation for primary prevention of cancer: meta-analysis of randomized controlled trials. J Community Hosp Intern Med Perspect. 2019;9(6):480-488. https:///doi.org/10.1080/20009666.2019.1701839
11. Hernández-Alonso P, Boughanem H, Canudas S, Becerra-Tomás N, Fernández de la Puente M, Babio N, et al. Circulating vitamin D levels and colorectal cancer risk: A meta-analysis and systematic review of case-control and prospective cohort studies. Crit Rev Food Sci Nutr. 2023;63(1):1-17. https://doi.org/10.1080/10408398.2021.1939649
12. Huncharek M, Muscat J, Kupelnick B. Colorectal cancer risk and dietary intake of calcium, vitamin D, and dairy products: A meta-analysis of 26,335 cases from 60 observational studies. Nutr Cancer. 2009;61(1):47-69. https://doi.org/10.1080/01635580802395733
13. Keum N, Giovannucci E. Vitamin D supplements and cancer incidence and mortality: A meta-analysis. Br J Cancer. 2014;111(5):976-80. https://doi.org/10.1038/bjc.2014.294
14. Khayatzadeh S, Feizi A, Saneei P, Esmaillzadeh A. Vitamin D intake, serum Vitamin D levels, and risk of gastric cancer: A systematic review and meta-analysis. J Res Med Sci. 2015;20(8):790-6. https://doi.org/10.4103/1735-1995.168404
15. Kim Y, Je Y. Vitamin D intake, blood 25(OH)D levels, and breast cancer risk or mortality: A meta-analysis. Br J Cancer. 2014;110(11):2772-84. https://doi.org/10.1038/bjc.2014.175
16. Lee JE, Li H, Chan AT, Hollis BW, Lee IM, Stampfer MJ, et al. Circulating levels of vitamin D and colon and rectal cancer: the Physicians' Health Study and a meta-analysis of prospective studies. Cancer Prev Res (Phila). 2011;4(5):735-43. https://doi.org10.1158/1940-6207.CAPR-10-0289
17. Liao MQ, Gao XP, Yu XX, Zeng YF, Li SN, Naicker N, et al. Effects of dairy products, calcium and vitamin D on ovarian cancer risk: a meta-analysis of twenty-nine epidemiological studies. Br J Nutr. 2020;124(10):1001-1012. https://doi.org/10.1017/S0007114520001075
18. Liao Y, Huang JL, Qiu MX, Ma ZW. Impact of serum vitamin D level on risk of bladder cancer: a systemic review and meta-analysis. Tumour Biol. 2015;36(3):1567-72. https://doi.org/10.1007/s13277-014-2728-9

19. Liu J, Dong Y, Lu C, Wang Y, Peng L, Jiang M, et al. Meta-analysis of the correlation between vitamin D and lung cancer risk and outcomes. Oncotarget. 2017;8(46):81040-81051. https://doi.org/10.18632/oncotarget.18766
20. Liu Y, Wang X, Sun X, Lu S, Liu S. Vitamin intake and pancreatic cancer risk reduction: A meta-analysis of observational studies. Medicine (Baltimore). 2018;97(13):e0114. https://doi.org/10.1097/MD.0000000000010114
21. Liu Y, Yu Q, Zhu Z, Zhang J, Chen M, Tang P, et al. Vitamin and multiple-vitamin supplement intake and incidence of colorectal cancer: a meta-analysis of cohort studies. Med Oncol. 2015;32(1):1-10. https://doi.org/10.1007/s12032-014-0434-5
22. Lopez-Caleya JF, Ortega-Valín L, Fernández-Villa T, Delgado-Rodríguez M, Martín-Sánchez V, Molina AJ. The role of calcium and vitamin D dietary intake on risk of colorectal cancer: systematic review and meta-analysis of case-control studies. Cancer Causes Control. 2022;33(2):167-182. https://doi.org/10.1007/s10552-021-01512-3
23. Maalmi H, Ordóñez-Mena JM, Schöttker B, Brenner H. Serum 25-hydroxyvitamin D levels and survival in colorectal and breast cancer patients: Systematic review and meta-analysis of prospective cohort studies. Eur J Cancer. 2014;50(8):1510-21. https://doi.org/10.1016/j.ejca.2014.02.006
24. Maalmi H, Walter V, Jansen L, Boakye D, Schöttker B, Hoffmeister M, et al. Association between blood 25-hydroxyvitamin D levels and survival in colorectal cancer patients: An updated systematic review and meta-analysis. Nutrients. 2018;10(7). https://doi.org/10.3390/nu10070896
25. Pu Y, Zhu G, Xu Y, Zheng S, Tang B, Huang H, et al. Association Between Vitamin D Exposure and Head and Neck Cancer: A Systematic Review With Meta-Analysis. Front Immunol. 2021;12(February):1-11. https://doi.org/10.3389/fimmu.2021.627226
26. Shahvazi S, Soltani S, Ahmadi SM, de Souza RJ, Salehi-Abargouei A. The Effect of Vitamin D Supplementation on Prostate Cancer: A Systematic Review and Meta-Analysis of Clinical Trials. Horm Metab Res. 2019;51(1):11-21. https://doi.org/10.1055/a-0774-8809
27. Sun K, Zuo M, Zhang Q, Wang K, Huang D, Zhang H. Anti-Tumor Effect of Vitamin D Combined with Calcium on Lung Cancer: A Systematic Review and Meta-Analysis. Nutr Cancer. 2021;73(11-12):2633-2642. https://doi.org/10.1080/01635581.2020.1850812
28. Wei H, Jing H, Wei Q, Wei G, Heng Z. Associations of the risk of lung cancer with serum 25-hydroxyvitamin D level and dietary vitamin D intake: A dose-response PRISMA meta-

analysis. Medicine (Baltimore). 2018;97(37):e12282. https://doi.org/10.1097/MD.0000000000012282
29. Xu Y, Qian M, Hong J, Ng DM, Yang T, Xu L, Ye X. The effect of vitamin D on the occurrence and development of colorectal cancer: a systematic review and meta-analysis. Int J Colorectal Dis. 2021;36(7):1329-1344. https://doi.org/10.1007/s00384-021-03879-w
30. Zhang R, Zhang Y, Liu Z, Pei Y, Xu P, Chong W, et al. Association between Vitamin D Supplementation and Cancer Mortality: A Systematic Review and Meta-Analysis. Cancers (Basel). 2022;14(15):3717. https://doi.org/10.3390/cancers14153717
31. Zhang X, Huang XZ, Chen WJ, Wu J, Chen Y, Wu CC, et al. Plasma 25-hydroxyvitamin D levels, vitamin D intake, and pancreatic cancer risk or mortality: A meta-analysis. Oncotarget. 2017;8(38):64395-406. https://doi.org/10.18632/oncotarget.18888
32. Zhang X, Niu W. Meta-analysis of randomized controlled trials on vitamin D supplement and cancer incidence and mortality. Biosci Rep. 2019;39(11):BSR20190369. https://doi.org/10.1042/BSR20190369
33. Zhang Y, Jiang X, Li X, Găman MA, Kord-Varkaneh H, Rahmani J, et al. Serum Vitamin D Levels and Risk of Liver Cancer: A Systematic Review and Dose-Response Meta-Analysis of Cohort Studies. Nutr Cancer. 2021;73(8):1-9. https://doi.org/10.1080/01635581.2020.1797127
34. Zhao Y, Chen C, Pan W, Gao M, He W, Mao R, et al. Comparative efficacy of vitamin D status in reducing the risk of bladder cancer: A systematic review and network meta-analysis. Nutrition. 2016;32(5):515-23. https://doi.org/10.1016/j.nut.2015.10.023
35. Zhou L, Chen B, Sheng L, Turner A. The effect of vitamin D supplementation on the risk of breast cancer: a trial sequential meta-analysis. Breast Cancer Res Treat. 2020;182(1):1-8. https://doi.org/10.1007/s10549-020-05669-4
36. Liu X, Zhou Y, Zou X. Correlation between Serum 25-Hydroxyvitamin D Levels and Gastric Cancer: A Systematic Review and Meta-Analysis. Curr Oncol. 2022;29(11):8390-8400. https://doi.org/10.3390/curroncol29110661
37. Choi YJ, Kim YH, Cho CH, Kim SH, Lee JE. Circulating levels of vitamin D and colorectal adenoma: A case-control study and a meta-analysis. World J Gastroenterol. 2015;21(29):8868-77. https://doi.org/10.3748/wjg.v21.i29.8868
38. Emmanouilidou G, Kalopitas G, Bakaloudi DR, Karanika E, Theocharidou E, Germanidis G, et al. Vitamin D as a chemopreventive agent in colorectal neoplasms. A systematic review and meta-analysis of randomized controlled trials. Pharmacol Ther. 2022;237:108252. https://doi.org/10.1016/j.pharmthera.2022.108252

39. Huang D, Lei S, Wu Y, Weng M, Zhou Y, Xu J, et al. Additively protective effects of vitamin D and calcium against colorectal adenoma incidence, malignant transformation and progression: A systematic review and meta-analysis. Clin Nutr. 2020;39(8):2525-2538. https://doi.org/10.1016/j.clnu.2019.11.012
40. Keum N, Chen QY, Lee DH, Manson JE, Giovannucci E. Vitamin D supplementation and total cancer incidence and mortality by daily vs. infrequent large-bolus dosing strategies: a meta-analysis of randomised controlled trials. Br J Cancer. 2022 Sep;127(5):872-878. https://doi.org/10.1038/s41416-022-01850-2
41. Song D, Deng Y, Liu K, Zhou L, Li N, Zheng Y, et al. Vitamin D intake, blood vitamin D levels, and the risk of breast cancer: a dose-response meta-analysis of observational studies. Aging (Albany NY). 2019;11(24):12708-12732. https://doi.org/10.18632/aging.102597
42. Wei MY, Garland CF, Gorham ED, Mohr SB, Giovannucci E. Vitamin D and prevention of colorectal adenoma: a meta-analysis. Cancer Epidemiol Biomarkers Prev. 2008;17(11):2958-69. https://doi.org/10.1158/1055-9965.EPI-08-0402
